# The deubiquitinase USP24 suppresses ferroptosis in triple-negative breast cancer by stabilizing DHODH protein

**DOI:** 10.1038/s41419-025-07895-4

**Published:** 2025-07-26

**Authors:** Li Yang, Xiaoqin An, Shangzhu Yang, Xiaowen Lin, Ziyuan Chen, Qian Xue, Xi Chen, Yuan Wang, Ding Yan, Shirui Chen, Yuqing Fan, Daolin Tang, Wenfeng Yu, Jinbao Liu, Xin Chen

**Affiliations:** 1 Department of Physiology, School of Basic Medical Sciences, Guizhou Medical University, Anshun, Guizhou China; 2 Provincial Key Laboratory of Medical Molecular Biology, Guizhou Medical University, Anshun, Guizhou China; 3 Key Laboratory of Biological Targeting Diagnosis, Therapy and Rehabilitation of Guangdong Higher Education Institutes, The Fifth Affiliated Hospital, Guangzhou Medical University, Guangzhou, Guangdong China; 4 Guangzhou Municipal and Guangdong Provincial Key Laboratory of Protein Modification and Disease, School of Basic Medical Sciences, Guangzhou Medical University, Guangzhou, Guangdong China; 5 Affiliated Cancer Hospital & Institute of Guangzhou Medical University, State Key Laboratory of Respiratory Disease, Guangzhou Medical University, Guangzhou, Guangdong China; 6 Department of Surgery, UT Southwestern Medical Center, Dallas, TX USA; 7 Key Laboratory of Endemic and Ethnic Diseases, Ministry of Education, School of Basic Medical Science, Guizhou Medical University, Guiyang, Guizhou China

**Keywords:** Cell death, Breast cancer

## Abstract

Triple-negative breast cancer (TNBC) is an aggressive subtype of invasive breast cancer characterized by limited treatment options and a poor prognosis. While ferroptosis, an iron-dependent form of regulated cell death, plays a role in tumor suppression, its specific molecular mechanisms in TNBC remain largely unexplored. In this study, we identify deubiquitinase USP24 as the most significantly altered enzyme among key deubiquitinating enzymes during ferroptosis in human TNBC cells. Silencing USP24 enhances ferroptosis-mediated tumor suppression in TNBC cells. Mechanistically, USP24 interacts directly with dihydroorotate dehydrogenase (DHODH) and deubiquitinates it, a process critical for maintaining coenzyme Q reduction and protecting cells from lipid peroxidation. Consistently, pharmacological inhibition of USP24 synergizes strongly with ferroptosis inducers in both in vitro and in vivo models via a DHODH-dependent pathway. These findings highlight USP24 as a potential therapeutic target to enhance ferroptosis sensitivity in TNBC.

## Introduction

Breast cancer is a leading cause of morbidity and mortality among women worldwide [1]. Based on molecular and histological evidence, breast cancer is classified into four major subtypes: Luminal A (ER^+^, PR^+^, HER2^−^), Luminal B (ER^+^, PR^+^ HER2^+^), HER2 enriched (ER^−^, PR^−^, HER2^+^), and basal-like/triple-negative breast cancer (TNBC) [2, 3]. Among these subtypes, TNBC is the most aggressive and metastatic, characterized by a poor prognosis, high recurrence rates, and elevated mortality [4]. TNBC presents unique therapeutic challenges due to its lack of established molecular targets and its malignant biological behavior [5, 6]. Moreover, TNBC is clinically and molecularly heterogeneous, comprising diverse subtypes with distinct biological characteristics, therapeutic responses, and clinical outcomes [6, 7]. These factors collectively make TNBC a formidable challenge in breast cancer management.

Ferroptosis is an iron-dependent cell death driven by the accumulation of ROS and lipid peroxidation products [8–10]. Morphologically, ferroptosis is characterized by necrotic features, including disrupted membrane integrity and increased membrane permeability, resulting from lipid peroxidation [11, 12]. This process has been implicated in various diseases, including ischemic reperfusion injury (IRI), neurodegenerative diseases, acute pancreatitis, acute kidney injury, liver fibrosis, stroke, diabetes, and atherosclerosis [13]. Additionally, ferroptosis has been observed in several cancers, including breast cancer, lung cancer, prostate cancer, neuroblastoma, and pancreatic cancer, offering a novel opportunity for cancer therapy [14–19]. Notably, ferroptosis inducers such as erastin and RSL3 have shown preclinical efficacy in TNBC by targeting glutathione (GSH) antioxidant system regulators, solute carrier family 7 member 11 (SLC7A11) and glutathione peroxidase 4 (GPX4), respectively [20]. However, poor pharmacological properties pose major challenges to their clinical translation [21–23]. These compounds augment lipid peroxidation to eliminate TNBC cells and can be combined with existing treatments (e.g., chemotherapy or immune checkpoint inhibitors) to overcome drug resistance [24]. Despite the therapeutic promise of ferroptosis, the underlying mechanisms regulating this process in TNBC remain poorly understood.

Dihydroorotate dehydrogenase (DHODH) is a flavin-dependent mitochondrial enzyme that catalyzes the conversion of dihydroorotate to orotate, a crucial step in de novo pyrimidine biosynthesis essential for DNA and RNA synthesis [25]. Given its role in cellular proliferation, DHODH is emerging as a promising therapeutic target in cancer [26]. Mitochondrial-localized DHODH also mediates ferroptosis resistance [27]. Mechanistically, DHODH limits mitochondrial lipid peroxidation by reducing ubiquinone (coenzyme Q, CoQ) to ubiquinol (CoQH₂), a potent inhibitor of lipid peroxidation [27]. Inhibiting DHODH has been proposed as a potential strategy to counteract ferroptosis resistance in cancer cells [28]. However, the mechanisms underlying the protein quality control of DHODH remain largely unclear.

Ubiquitination, a critical post-translational modification (PTM), plays an essential role in maintaining protein stability and functionality through the coordinated actions of ubiquitin E3 ligases and deubiquitinases (DUBs) [29]. This reversible modification is essential for protein quality control, and its dysregulation can impair protein stability, leading to cancer progression [30]. Alterations in protein ubiquitination are prevalent in various cancers, and small-molecule inhibitors targeting this process have shown promise as potential cancer therapies [31]. Among DUBs, ubiquitin-specific peptidases (USPs) are significant regulators of cancer progression [32, 33]. USP24, a member of DUBs, is located at the PARK10 locus on chromosome 1 and was initially associated with Parkinson’s disease [34, 35]. By cleaving ubiquitin from substrates, USP24 influences the stability, localization, and interactions of various proteins, including PLK1, TRAF2, Beclin1, and GSDMB, in cancers such as gastric carcinoma, hepatocellular carcinoma, and bladder cancer [36–38]. Through these interactions, USP24 is implicated in processes such as cell proliferation, glycolysis, apoptosis, and immune evasion. However, its role in TNBC progression remains poorly understood.

Recent studies have highlighted the multifaceted role of DUBs in modulating ferroptosis through the stabilization and regulation of key ferroptotic proteins, thus influencing cancer progression and therapeutic responses. For instance, USP52 and OTUD5 prevent ferroptosis by stabilizing SLC7A11, thereby promoting the progression of bladder cancer and TNBC, respectively [39, 40]. Similarly, USP8 plays a pivotal role in modulating the O-GlcNAcylation of SLC7A11 by stabilizing the OGT enzyme, thereby suppressing ferroptosis in hepatocellular carcinoma [41]. USP8 also contributes to the regulation of ferroptosis by maintaining GPX4 homeostasis, thereby influencing cellular responses to cancer immunotherapy [42]. USP35 has been shown to regulate ferroptosis susceptibility in lung cancer by targeting ferroportin, a protein involved in iron export [43]. Furthermore, USP24 inhibition induces ferroptosis in drug-resistant lung and brain cancer cells by elevating ACSL4 levels, activating autophagy, and facilitating GPX4 degradation [44]. Collectively, these studies suggest that DUBs play a critical role in ferroptosis regulation across various cancer types. Targeting DUBs, therefore, presents a promising therapeutic strategy to modulate ferroptosis in cancer and enhance treatment efficacy.

In this study, we identify USP24 as a novel suppressor of ferroptosis in TNBC. We demonstrate that USP24 functions as a DUB for DHODH, enhancing DHODH stability by removing its ubiquitination both in vitro and in vivo. This interaction prevents ferroptosis by maintaining DHODH-mediated reduction of ubiquinone, which is essential for mitigating lipid peroxidation. Furthermore, silencing or pharmacological inhibition of USP24 sensitizes TNBC cells to ferroptosis. Our findings highlight USP24 as a potential therapeutic target to modulate ferroptosis and offer a promising strategy for improving treatment outcomes in TNBC.

## Materials and methods

### Cell lines and cell culture

The MDA-MB-231 and MDA-MB-468 human TNBC cell lines were purchased from the American Type Culture Collection (ATCC; Manassas VA, USA). The MDA-MB-231 and MDA-MB-468 cells were cultured in DMEM/F12 medium supplemented with 10% fetal bovine serum (FBS). These cell lines were grown in an incubator maintained at 37°C with an atmosphere of 5% CO_2_. All the cells were mycoplasma-free and authenticated using short tandem repeat (STR) DNA profiling analysis.

### Reagents and primary antibodies

Erastin (S7242), RSL3 (S8155), ML210 (23282), FIN56 (S8254), ferrostain-1 (S7243), Z-VAD-FMK (Z-VAD, S7023), chloroquine (CQ, S6999), WP1130 (S2243), cycloheximide (S7418), and MG132 (S2619) were purchased from Selleck Chem (Houston, TX, USA). ML162 (SML2561) was purchased from Sigma Aldrich. MitoQH2 (89950) was purchased from Cayman Chemical. USP7 (ab108931), USP16 (ab236628), USP24 (ab72241), USP35 (ab254939, USP39 (ab131244), ALOX12 (ab211506), 4-HNE (ab48506), and GPX4 (ab125066) antibodies were purchased from Abcam (USA). USP5 (10473-1-AP), USP9X (81892-1-RR), USP10 (19374-1-AP), USP13 (16840-1-AP), USP24 (13126-1-AP), USP28 (17707-1-AP), USP38 (17767-1-AP), USP48 (12076-1-AP), USP53 (83846-1-RR), DHODH (14877-1-AP), GSS (15712-1-AP), ACSL4 (22401-1-AP), NRF2 (80593-1-AP), Ki67 (27309-1-AP), and FSP1 (20886-1-AP) antibodies were purchased from Proteintech (Chicago, USA). USP12 (DF9981) antibodies were purchased from Affinity Biosciences. USP30 (C117066) antibodies were purchased from Sigma Aldrich. USP14 (119313), SLC7A11 (98051), cleaved caspase-3 (9661S), and Flag (8146S) antibodies were purchased from Cell Signaling Technology (Beverly, MA, USA). Ubiquitin (sc-8017) and p53 (9282S), antibodies were purchased from Santa Cruz Biotechnology (CA, USA). Ferritin (BS90500) antibody was purchased from Bioworld Technology (Louis Park, MN, USA). DHODH (M04035) antibody was purchased from BOSTER Biological Technology.

### Cell viability assay

Cell viability was analyzed with a Cell Counting Kit-8 (CCK8; DOJINDO, CK04). Cells were seeded in a 96-well plate at a density of 1 × 10^4^ cells per well and exposed to the indicated drug. Subsequently, 100 μl of fresh culture medium containing 10 μl of CCK-8 solution was introduced to the cells. The cells were then incubated in a 37 °C incubator with 5% CO_2_ for 1 h. The absorbance was measured at a wavelength of 450 nm using a microplate reader. (Thermo Scientific, Varioskan Flash).

### Lentiviral shRNA transfection

Lentivirus vector (pLKO.1-U6-EF1a-copGFP-T2A-puro) containing shRNAs targeting USP24 or nonspecific sequences (control shRNAs) was purchased from Guangzhou IGE Biotechnology Co., Ltd. The sequences of the USP24 shRNAs were as follows (all are shown in 5′–3′ orientation): USP24 shRNA-1: CCGGCTCTCGTATGTAACGTATTTGCTCGAGCAAATACGTTACATACGAGAGTTTTTGAATT; USP24 shRNA-2: CCGGACAATACTGTGACCGTATAAACTCGAGTTTATACGGTCACAGTATTGTTTTTTGAATT. Lentivirus vectors (pRRLSIN-cPPT-U6-shRNA-SFFV-EGFP-SV40-puro) containing shRNAs targeting USP9X or nonspecific sequences (control shRNAs) were purchased from Shanghai Genechem Co., Ltd. The target sequences of the USP9X shRNAs were as follows: USP9X shRNA-1:GGTCGTTACAGCTAGTATTTTA; USP9X shRNA-2: CGACCCTAAACGTAGACATTA. The cells were plated at a density of 3 × 10^5^ cells/well and cultured for 24 h. Then, lentivirus vectors expressing shRNAs were added to the cells at a multiplicity of infection of 15. After transfection for 48 h, we conducted selection with puromycin (Selleck Chem, S7417) at a concentration of 5 µg/ ml to obtain successfully transfected cells.

### Plasmids and transfection

The plasmid (pCMV-MCS-3FLAG) carrying the full-length human DHODH CDS (gene ID: NM_001361.5) was purchased from Mailgene Biosciences Co., Ltd. (Guangzhou, China). The transfection of plasmid was performed with Lipofectamine 3000 (Thermo Fisher Scientific, L3000-015) according to the manufacturer’s protocol. respectively.

### Lipid peroxidation assay

Lipid peroxidation was analyzed by Liperfluo (DOJINDO, L248) staining. Cells were seeded into 6-well plates at a density of 1 × 10^5^ cells and incubated with the indicated treatment in a 37 °C, 5% CO_2_ incubator. During the last 30 min of incubation, 1.5 μM Liperfluo and Hoechst 33342 were added. The cells were then imaged using a fluorescence microscope (ZEISS, Axio Observer 5) and the relative fluorescence intensity was quantified as the ratio of green fluorescence intensity (Liperfluo) to blue fluorescence intensity (Hoechst 33342).

### Cell death assay

Cell death was analyzed using propidium iodide (PI) staining methods. Cells were seeded in 6-well plates at a density of 2 × 10^5^ cells/well. The next day, cells were incubated with the specified treatment. Then, the cells were stained with 1.5 μM PI for 30 min in a 37 °C, 5% CO_2_ incubator. PI-positive cells were imaged using a fluorescence microscope (ZEISS, Axio Observer 5) and the relative proportion of PI-positive cells was quantified.

### 3D spheroids cell culture assay

MDA-MB-231 cells were seeded on 96-well round-bottom ultra-low attachment plates (Corning costar 7007) at a density of 2 × 10^3^ cells per well. After 48 h of growth, the spheres were treated with the indicated treatment and incubated for another 48 h. Propidium iodide (PI; 1.5 μM) and Hoechst 33342 (diluted to a 1:1000 ratio) were directly added to the wells and incubated for 30 min. Subsequently, images were captured using a fluorescence microscope (ZEISS, Axio Observer 5). The relative PI fluorescence intensity was quantified as the ratio of red fluorescence intensity (PI) to blue fluorescence intensity (Hoechst 33342).

### Western blotting analysis

Cells were lysed on ice with cell lysis buffer (Beyotime, P0013) containing protease inhibitors (Sigma-Aldrich, P8340), and then incubated on ice for 10 min. BCA assay (Thermo Fisher Scientific, 23225) was used to determine the protein concentration of each sample (20–40 μg), which was separated by SDS-PAGE using 10% sodium dodecyl sulfate polyacrylamide gel electrophoresis (SDS-PAGE) and transferred to a polyvinylidene fluoride (PVDF) membrane. The membrane was blocked with 5% non-fat dry milk in TBS with Tween 20 (TBST) for 1 hour and incubated with primary antibody overnight at 4 °C. After washing three times with TBST, the membrane was incubated at room temperature for 1 h with goat anti-rabbit or anti-mouse IgG HRP-linked secondary antibody (Cell Signaling Technology, 7076S or 7074S), followed by washing with PBST. The western blotting luminol reagents (Santa Cruz Biotechnology, sc-2048) were used to measure chemiluminescent signals on the PVDF membrane.

### Cloning formation

Cells were seeded into 6-well plates, ensuring consistency in cell count across each well. Then, the cells were placed in a cell culture incubator and allowed to proliferate for a period of 10–14 days. After that, the 6-well plates were washed with PBS, fixed with 4% paraformaldehyde for 15–30 min, and then washed with PBS to remove excess fixative. Crystal violet staining was applied for 15–20 min, followed by rinsing with tap water to remove background staining.

### Transwell assay

Cells were seeded at a density of 2 × 10^5^ cells/well and cultured for 24 h. A total of 0.5–1 × 10^5^ serum-free medium was co-seeded in the upper chamber of the Transwell insert (Corning, 3422), while 10% FBS medium was added to the lower chamber. After 24 h of incubation, the cells were washed with PBS and fixed with 4% paraformaldehyde. Non-migratory cells on the upper side of the Transwell insert were wiped off with a cotton swab. The migratory cells were stained with 1% crystal violet for 5–10 min and quantified.

### Immunoprecipitation analysis

Cells were lysed in low-temperature cell lysis buffer (Beyotime, P0013) at 4 °C, followed by centrifugation at 13,000 × *g* for 15 min. The concentration of protein in the supernatant was determined using the BCA assay. Prior to immunoprecipitation, samples containing equal amounts of protein were pre-cleared with Dynabeads (Thermo Fisher Scientific, 14311D) at 4 °C for 3 h. Then, these samples were incubated in the presence of Dynabeads with various unrelated IgG or specific antibodies (5 μg/ml) for 2 h, followed by gently shaking overnight at 4 °C to ensure thorough interaction. Following incubation, the Dynabeads were washed with PBS, and proteins were eluted using sample buffer (Cell Signaling Technology, 56036S) and boiled before SDS-PAGE analysis.

### Immunofluorescence assay

Cells were fixed with 4% paraformaldehyde (Servicebio, G1101) and permeabilized with 0.3% Triton X-100 (Solarbio, T8200). The cells were then incubated overnight at 4 °C with primary antibody and 1% bovine serum albumin (Sigma-Aldrich, 9048-46-8) in PBS, followed by three washes with PBS and application of secondary antibody. Cells were counterstained with 4′,6-diamidino-2-phenylindole (DAPI; Abcam, ab104139) for 10 min. After three washes with PBS, the slides were protected with coverslips and sealed with a mounting medium containing nail polish to prevent fading. Immunofluorescence images were obtained using a confocal laser scanning microscope (Leica DLS).

### Quantitative RT-PCR (qPCR) analysis

Total RNA was extracted and purified from cultured cells using the standard TRIZOL RNA extraction protocol (AG, AG21102). First-strand cDNA was synthesized from 1 µg RNA using the Evo M-MLV RT Kit and the gDNA Clean for qPCR Kit (Accurate Biology, AG11705). Briefly, a total of 10 µl reaction mixture, 1 µl of Evo M-MLV RTase Enzyme mix, 1 µl of RT primer mix, 4 µl of 5× RTase reaction Buffer mix I, and 4 µl of RNA-free water were mixed to prepare a 20 µl reaction. SYBR Green Premix Pro Taq HS qPCR Kit (Accurate Biology, AG11701) was used for real-time quantitative PCR amplification of cDNA from different cell samples with specific primers. Gene expression levels were calculated using the 2 − ΔΔCt method and normalized to RNA18S. The relative concentration of mRNA was expressed in arbitrary units with the untreated group assigned a value of 1. The primers, which were synthesized and desalted from Servicebio. The primers of USP24: F: 5′CAGTTGTGCTCTCCTGTGGA 3’; R: 5′AGGGATTTCTCCTGCTCCAT3’. The primers of DHODH: F: 5′GAGGACATTGCCAGTGTGGTCA 3’; R: 5′TTCCCACTCAGCCCTCCTGTTT 3’. The primers of 18S: F: 5′AACCCGTTGAACCCCATT3’; R: 5′CCATCCAATCGGTAGTAGCG3’.

### Immunohistochemistry

Immunostaining of tumor xenograft sections was performed using the MaxVision Kit (G1004, Servicebio, Wuhan, China) according to the manufacturer’s instructions. Protein expression was detected with primary antibodies and analyzed using Image-Pro Plus 7 software. Relative staining intensity was normalized to the control group.

### Nude mouse xenograft model

The care and use of all animals in this study were conducted in accordance with the ethical treatment guidelines of the Guangdong Provincial Animal Center and were approved by the Animal Care and Use Committee of Guangzhou Medical University (approval number: GY2024-599). The xenograft model was established as previously described [45]. Four-week-old female nude BALB/c mice were purchased from Guangdong Vital River Laboratory Animal Technology Co., Ltd. and housed in the specific pathogen-free (SPF) Animal Center of Guangzhou Medical University. Mice were randomly divided into assigned to each group. MDA-MB-231 or MDA-MB-231 shUSP24 (#1) cells (2.5 × 10^6^) were subcutaneously injected into the axillary region of the nude mice in 200 µl PBS. Tumor size was recorded every other day starting 10 days post-inoculation. The tumor volume was determined using the formula: π/6 × length × width^2^. On day 12 post-treatment, tumor xenografts were harvested, weighed, and utilized for subsequent analyses.

### Statistical analysis

GraphPad Prism 10.8.1 was used to collect and analyze data. Data are presented as mean ± SD. A one-way or two-way analysis of variance (ANOVA) with Tukey’s multiple comparisons test was used for comparison among the different groups. *P* < 0.05 was considered statistically significant.

## Results

### USP24 is selectively downregulated in TNBC cells during ferroptosis

**Fig. 1:**
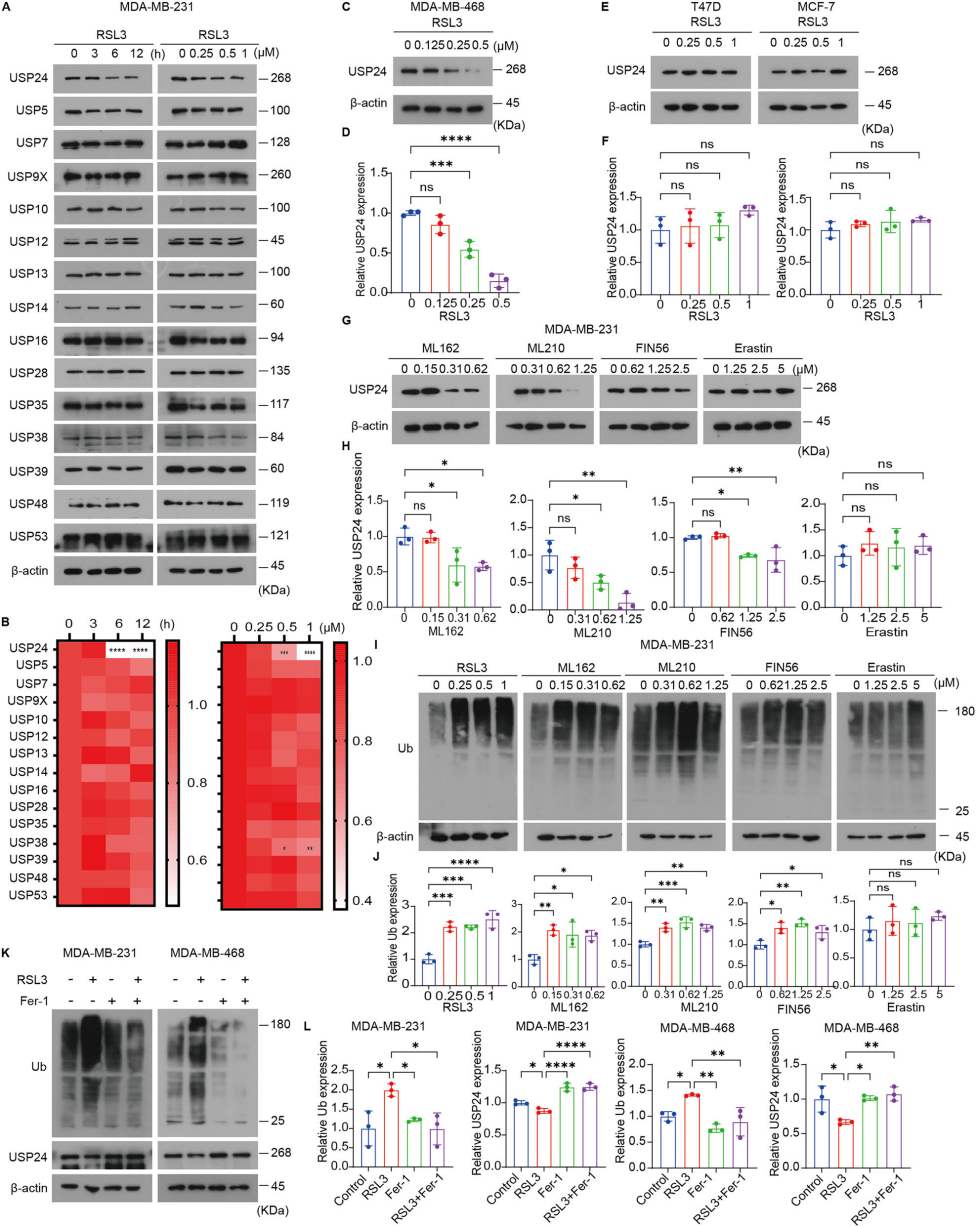
USP24 is downregulated during ferroptosis induced by GPX4 inhibitors. **A**, **B** MDA-MB-231 cells were exposed to 0.5 μM RSL3 for varying time intervals or to different concentrations of RSL3 for 12 h. Subsequently, cellular protein expression was assessed by western blot analysis. Quantification of protein levels, normalized to β-actin, was performed based on three independent experiments. Data were presented as Mean ± SD. *P < 0.05, **P < 0.01, ***P < 0.001, ****P < 0.0001. **C**, **D** MDA-MB-468 cells were treated with the indicated doses of RSL3 for 12 h, and then cellular proteins were analyzed with western blotting. Quantification of protein levels, normalized to β-actin, was performed based on three independent experiments. Data were presented as Mean ± SD, n = 3. ***P < 0.001, ****P < 0.0001, ns no significance. **E**, **F** T47D and MCF-7 cells were treated with the indicated doses of RSL3 for 12 h, and then cellular proteins were analyzed with western blotting. Quantification of protein levels, normalized to β-actin, was performed based on three independent experiments. Data were presented as Mean ± SD. ns no significance. **G**, **H** MDA-MB-231 cells were treated with the indicated concentration gradient of ferroptosis inducers, including RSL3, ML162, ML210, FIN56, and erastin for 12 h, and then USP24 proteins were analyzed with western blotting. Quantification of protein levels, normalized to β-actin, was performed based on three independent experiments. Data were presented as Mean ± SD. *P < 0.05, **P < 0.01. ns no significance. **I**, **J** MDA-MB-231 cells were treated with the indicated concentration gradient of ferroptosis inducers, including RSL3, ML162, ML210, FIN56, and erastin for 12 h, and then ubiquitinated proteins (Ub) were analyzed with western blotting. Quantification of protein levels, normalized to β-actin, was performed based on three independent experiments. Data were presented as Mean ± SD. *P < 0.05, **P < 0.01, ***P < 0. 001, ****P < 0.0001. ns no significance. **K**, **L** Western blotting was used to examine the effects of ferroptosis inhibitor ferrostatin-1 (Fer-1, 2 μM) on RSL3 (0.5 μM for MDA-MB-231 cells; 0.25 μM for MDA-MB-468 cells, 12 h)-induced ubiquitination (Ub) accumulation and USP24 downregulation. Quantification of protein levels, normalized to β-actin, was performed based on three independent experiments. Data were presented as Mean ± SD. *P < 0.05, **P < 0.01, ****P < 0.0001.

To investigate alterations in DUBs during the ferroptosis process, we examined the impact of RSL3 (a well-characterized GPX4 inhibitor [46]) on the protein levels of various DUBs in MDA-MB-231 TNBC cells. Western blot assay revealed that RSL3 obviously downregulated USP24 protein levels in a dose- and time-dependent manner (Fig. 1A, B). This effect was not limited to MDA-MB-231 cells, as similar results were observed in MDA-MB-468 TNBC cells, where RSL3 also induced a marked reduction in USP24 protein levels (Fig. 1C, D). RSL3 treatment also results in a decrease in USP38 levels in MDA-MB-468 cells, whereas it has no effect on USP38 expression in MDA-MB-231 cells, suggesting that USP38 regulation is cell-type dependent (Fig. 1A, B). However, the effects of RSL3 on other DUBs were relatively minor (Fig. 1A, B). Consistently, we found that RSL3 downregulated the gene expression of *USP24* in MDA-MB-231 and MDA-MB-468 cells (Supplementary Fig. 1A). We also examined the effect of RSL3 on USP24 protein expression in non-TNBC cells, such as T47D and MCF-7. The results showed that RSL3 did not alter USP24 expression in these breast cancer cells, suggesting that ferroptosis-induced USP24 downregulation is cell-type selective (Fig. 1E, F).

To determine whether this effect was specific to RSL3, we assessed the influence of other ferroptosis inducers on USP24 expression. Similar to RSL3, additional GPX4 inhibitors, including ML162, ML210, and FIN56 [47, 48], consistently downregulated USP24 gene and protein levels (Fig. 1G, H and Supplementary Fig. 1B). In contrast, erastin, an inhibitor of the SLC7A11 [8], had no effect on USP24 protein levels and only a minimal impact on its gene expression (Fig. 1G, H and Supplementary Fig. 1B). These findings suggest that GPX4 inhibition, rather than SLC7A11 blockade, is specifically linked to the downregulation of USP24.

Given the key regulatory role of DUBs in ubiquitination, we further evaluated the effects of GPX4 inhibitors on ubiquitination dynamics in MDA-MB-231 cells. Treatment with RSL3, ML162, ML210, or FIN56 resulted in a significant accumulation of ubiquitinated proteins, whereas erastin failed to induce this effect (Fig. 1I, J). To confirm the relationship between ferroptosis and these observations, we tested the combination of GPX4 inhibitors with ferrostatin-1, a potent ferroptosis inhibitor. Ferrostatin-1 completely reversed the ubiquitination accumulation and USP24 downregulation induced by GPX4 inhibitors (Fig. 1K, L and Supplementary Fig. 1C).

Collectively, these findings demonstrate that GPX4 inhibitors induce downregulation of USP24 and promote ubiquitination accumulation in TNBC cells.

### Knockdown of USP24 promotes ferroptosis in TNBC cells

**Fig. 2:**
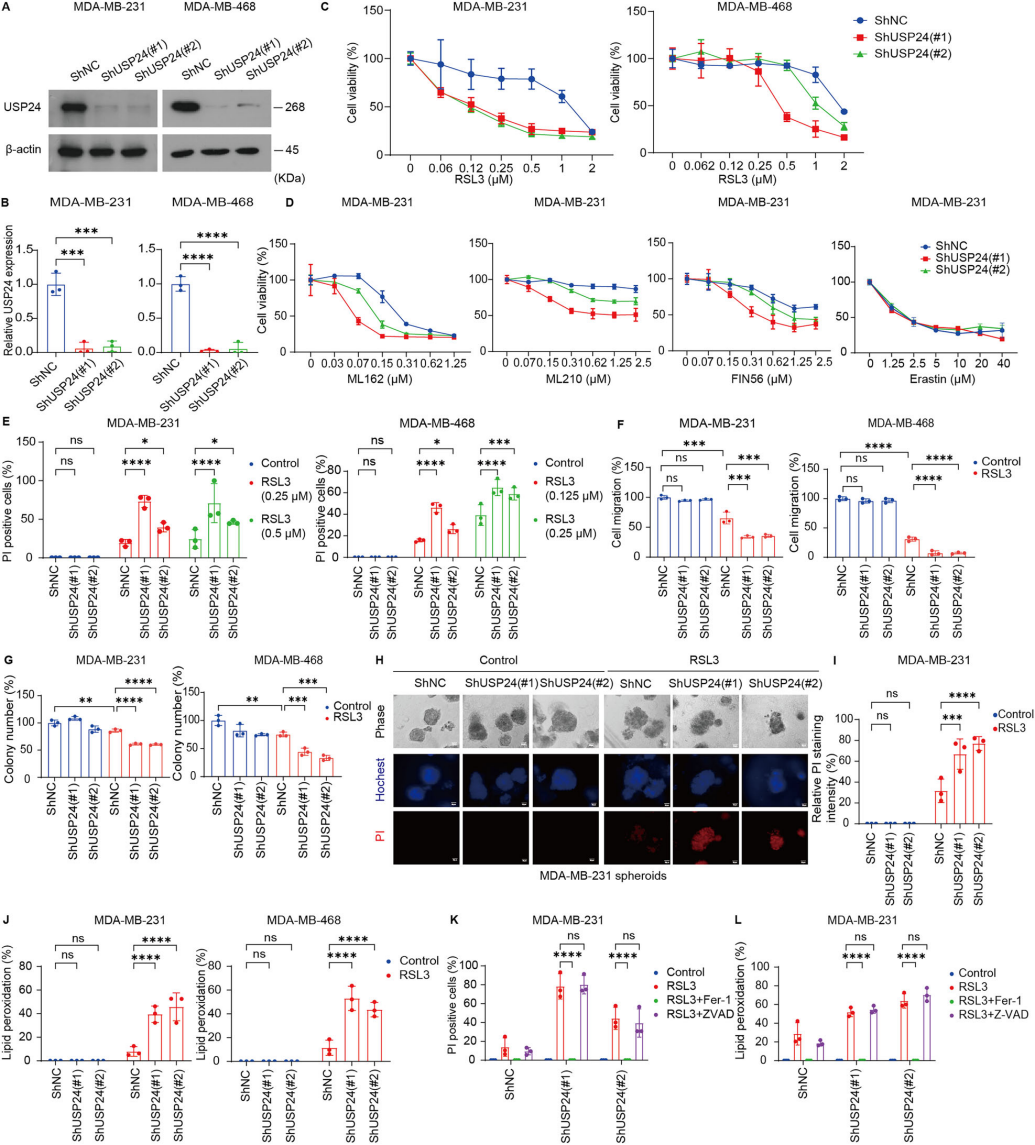
Knockdown of USP24 promotes ferroptosis in TNBC cells. **A**, **B** MDA-MB-231 and MDA-MB-468 cells were stably transfected with two individual USP24 shRNAs or control shRNA. USP24 expression in cells was measured by Western blotting. Quantification of protein levels, normalized to β-actin, was performed based on three independent experiments. Data were presented as Mean ± SD. ***P < 0.001, ****P < 0.0001. **C** Cell viability of the indicated MDA-MB-231 and MDA-MB-468 cell lines treated with the indicated doses of RSL3 for 24 h. **D** Cell viability of the indicated MDA-MB-231 cell lines treated with the indicated doses of ML162, ML210, FIN56, and erastin for 24 h. **E** Cell death was assessed using propidium iodide (PI) staining in the indicated MDA-MB-231 and MDA-MB-468 cell lines treated with the indicated doses of RSL3 for 24 h. Quantification of PI-positive cells was shown. Mean ± SD, n = 3. *P < 0.05, ***P < 0.001, ****P < 0.0001. ns no significance. Migration (**F**) and cloning formation (**G**) abilities of the indicated MDA-MB**-**231 and MDA-MB-468 cell lines treated with RSL3 (0.5 μM for MDA-MB-231 cells; 0.25 μM for MDA-MB-468 cells) for 6 h. Mean ± SD, n = 3. **P < 0.01, ***P < 0.001, ****P < 0.0001. ns no significance. **H**, **I** The representative phase-contrast and propidium iodide (PI) staining images of the indicated MDA-MB-231 three-dimensional spheroids treated with 0.5 μM RSL3 for 48 h. The relative PI fluorescence intensity was quantified as the ratio of red fluorescence intensity (PI) to blue fluorescence intensity (Hoechst 33342). Mean ± SD, n = 3. ***P < 0.001, ****P < 0.0001. ns no significance. **J** Lipid peroxidation of the indicated MDA-MB-231 and MDA-MB-468 cell lines treated with RSL3 (0.5 μM for MDA**-**MB**-**231 cells; 0.25 μM for MDA-MB-468 cells) for 6 h. Mean ± SD, n = 3. ****P < 0.0001. ns no significance. **K** Cell death was assessed using propidium iodide (PI) staining in the indicated MDA-MB-231 cell treated with 0.5 μM RSL3, in the presence of ferroptosis inhibitor ferrostain-1 (Fer-1, 2 μM) or apoptosis inhibitor Z-VAD-FMK (Z-AVD, 10 μM) for 24 h. Quantification of PI-positive cells was shown. Mean ± SD, n = 3. ****P < 0.0001. ns no significance. **L** Lipid peroxidation of the indicated MDA-MB-231 cell lines treated with 0.5 μM RSL3, in the presence of ferroptosis inhibitor ferrostain-1 (Fer-1, 2 μM) or apoptosis inhibitor Z-VAD-FMK (Z-AVD, 10 μM) for 6 h. Mean ± SD, n = 3. ****P < 0.0001. ns no significance.

The above findings suggest a potential role for USP24 in ferroptosis regulation. To validate this hypothesis, we established *USP24*-stable knockdown cell lines in MDA-MB-231 and MDA-MB-468 cells. Knockdown of *USP24* significantly increased the sensitivity of both cell lines to growth inhibition induced by RSL3 (Fig. 2A-C). Additionally, *USP24* knockdown enhanced the anti-tumor effects of other GPX4 inhibitors, including ML162, ML210, and FIN56, in MDA-MB-231 cells, while exerting minimal impact on erastin-induced ferroptosis (Fig. 2D).

To further confirm the role of USP24 in ferroptosis, we assessed cell death using propidium iodide (PI) staining. The results demonstrated that *USP24* knockdown significantly enhanced RSL3-induced cell death (Fig. 2E). Moreover, cell migration and colony formation assays revealed that *USP24* knockdown potentiated the anti-tumor effects of RSL3, reducing both migratory capacity (Fig. 2F and Supplementary Fig. 2) and colony formation (Fig. 2G) in TNBC cells. Previous studies have reported that USP24 inhibition suppresses the migration of lung cancer cells [49], while USP24 silencing promotes cell proliferation and migration in hepatocellular carcinoma [50]. The lack of a significant effect in our TNBC model suggests that the role of USP24 in cell migration may be context-dependent. To mimic the tumor microenvironment more closely, we utilized a 3D spheroid model of MDA-MB-231 cells. Consistent with 2D culture findings, *USP24* knockdown enhanced RSL3-induced cell death in the 3D spheroid model (Fig. 2H, I).

Lipid peroxidation is a hallmark of ferroptosis [8]. To investigate this, we measured lipid peroxidation levels using the Liperfluo probe. *USP24* knockdown increased RSL3-induced lipid peroxidation in TNBC cells (Fig. 2J). Importantly, treatment with the ferroptosis inhibitor ferrostatin-1 reversed the increased ferroptosis sensitivity and lipid peroxidation observed in USP24 knockdown cells, whereas the apoptosis inhibitor Z-VAD-FMK had no effect (Fig. 2K, L).

These findings collectively suggest that USP24 functions as an anti-ferroptotic protein in TNBC cells by modulating sensitivity to ferroptosis and lipid peroxidation.

### USP24 inhibition synergizes with ferroptosis inducers to enhance anti-tumor effects in TNBC cells

**Fig. 3:**
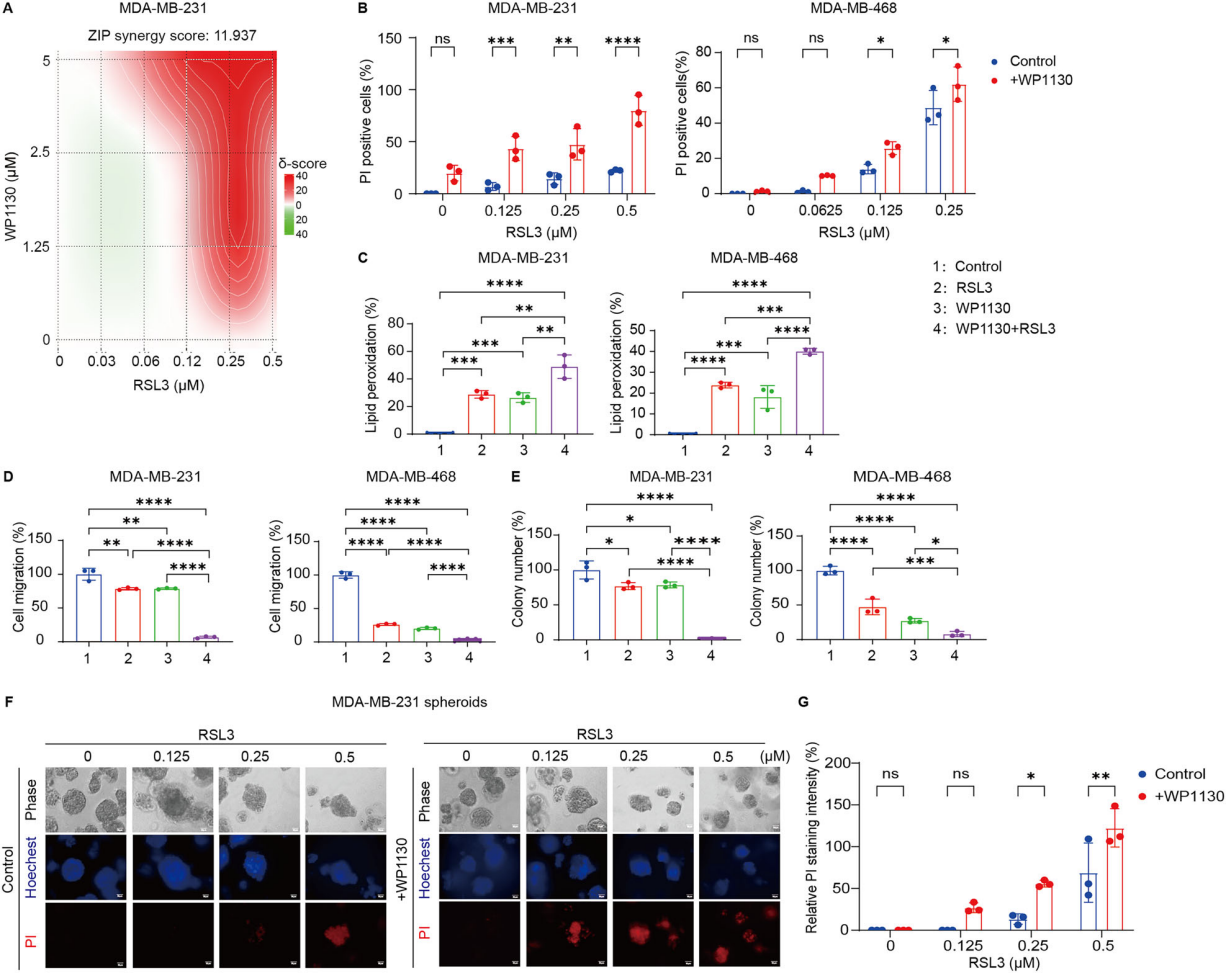
USP24 inhibitor exhibits a synergistic effect with ferroptosis inducer in TNBC cells. **A** Cell viability of MDA-MB-231 cells treated with the indicated concentrations of RSL3 in combination with WP1130, and the results was analyzed by SynergyFinder web-application. ZIP synergy score>10 indicates a synergistic effect between the two agents. **B** Cell death was assessed using propidium iodide (PI) staining in MDA-MB-231 and MDA-MB-468 cells treated with the indicated concentrations of RSL3 in the presence or absence of WP1130 (0.5 μM) for 24 h. Quantification of PI-positive cells was shown. Mean ± SD, n = 3. *P < 0.05, **P < 0.01, ***P < 0.001, ****P < 0.0001; ns no significance. **C** Lipid peroxidation of MDA-MB-231 and MDA-MB-468 cells treated with RSL3 (0.5 μM for MDA-MB-231 cells; 0.25 μM for MDA-MB-468 cells) in the presence or absence of WP1130 (0.5 μM) for 6 h. Mean ± SD, n = 3. **P < 0.01, ***P < 0.0001, ****P < 0.0001. Migration (**D**) and cloning formation (**E**) abilities of MDA-MB-231 cells and MDA-MB-468 cells treated with RSL3 (0.5 μM for MDA-MB-231 cells; 0.25 μM for MDA-MB-468 cells) in the presence or absence of WP1130 (0.5 μM) for 6 h. Mean ± SD, n = 3. *P < 0.05, **P < 0.01, ***P < 0.001, ****P < 0.0001. **F**, **G** The representative phase-contrast and propidium iodide (PI) staining images of MDA-MB-231 three-dimensional (3D) spheroids treated with the indicated doses of RSL3 in the presence or absence of WP1130 (0.5 μM) for 48 h. The relative PI fluorescence intensity was quantified as the ratio of red fluorescence intensity (PI) to blue fluorescence intensity (Hoechst 33342). Mean ± SD, n = 3. *P < 0.05, **P < 0.01. ns no significance.

To further evaluate the role of USP24 in ferroptosis, we utilized WP1130, a known USP24 inhibitor [51], to assess its impact on ferroptosis in TNBC cells. Treatment with WP1130 and the ferroptosis inducer RSL3 demonstrated a significant synergistic effect, with a ZIP synergy score of 11.937 in MDA-MB-231 cells (calculated using the SynergyFinder platform: https://synergyfinder.fimm.fi) (Fig. 3A).

PI staining and Liperfluo-based lipid peroxidation assays further revealed that WP1130 enhanced RSL3-induced cell death and lipid peroxidation in MDA-MB-231 cells (Fig. 3B, C). In addition, the combination of WP1130 and RSL3 inhibited TNBC cell migration (Fig. 3D and Supplementary Fig. 3) and reduced colony formation (Fig. 3E), as assessed by cell migration and colony formation assays. Consistent with the 2D culture findings, the combination of WP1130 and RSL3 also showed a marked enhancement of cytotoxic effects, significantly reducing spheroid size and increasing cell death (Fig. 3F, G).

These results provide evidence that USP24 inhibition via WP1130 potentiates ferroptosis in TNBC cells, reinforcing the therapeutic potential of targeting USP24 to enhance ferroptosis-driven anti-cancer strategies.

### DHODH is a substrate of USP24 in TNBC cells

**Fig. 4:**
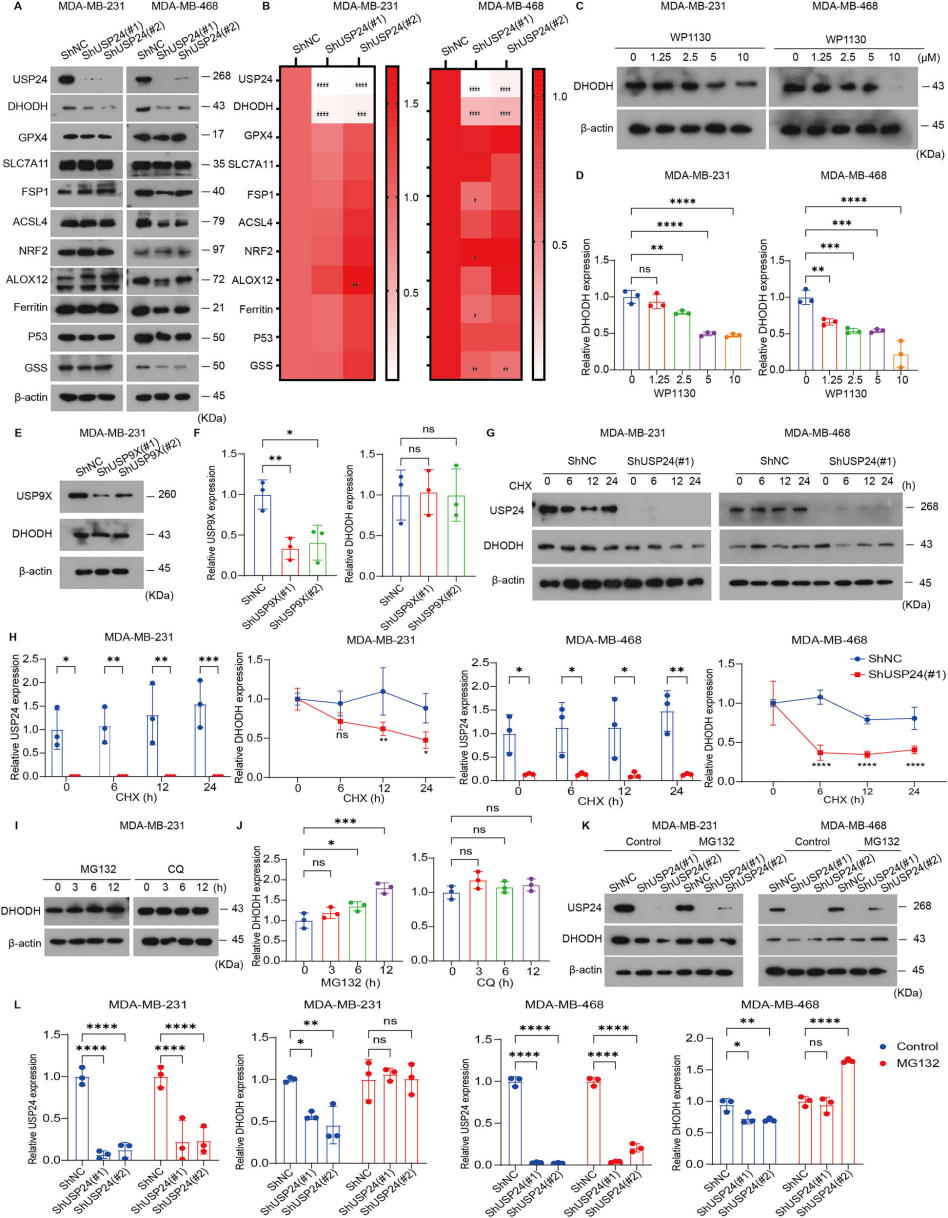
DHODH is a substrate of USP24 in TNBC cells. **A**, **B** Cellular proteins in the indicated MDA-MB-231 and MDA-MB-468 cell lines was measured by Western blotting. Quantification of protein levels, normalized to β-actin, was performed based on three independent experiments. Data were presented as Mean ± SD. *P < 0.05, **P < 0.01, ***P < 0.001, ****P < 0.0001. **C**, **D** Western blotting analysis of DHODH proteins in MDA-MB-231 and MDA-MB-468 cells treated with the indicated doses of WP1130 for 24 h. Quantification of protein levels, normalized to β-actin, was performed based on three independent experiments. Data were presented as Mean ± SD. **P < 0.01, ***P < 0.001, ****P < 0.0001. **E**, **F** MDA-MB-231 cells were stably transfected with two individual USP9X shRNAs or control shRNA. USP9X expression in cells was measured by Western blotting. Quantification of protein levels, normalized to β-actin, was performed based on three independent experiments. Data were presented as Mean ± SD. *P < 0.05, **P < 0.01. ns no significance. **G**, **H** Western blotting analysis of cellular proteins in the indicated MDA-MB-231 and MDA-MB-468 cell lines treated with cycloheximide (CHX, 100 μg/ml for MDA-MB-231 cells; 30 μg/ml for MDA-MB-468 cells) for the indicated time periods. Quantification of protein levels, normalized to β-actin, was performed based on three independent experiments. Data were presented as Mean ± SD. *P < 0.05, **P < 0.01, ***P < 0.001. ns no significance. **I**, **J** MDA-MB-231 cells were treated with MG132 (10 μM) or chloroquine (CQ, 30 μM) for the indicated time periods. The protein levels of DHODH were measured by Western blotting. Quantification of protein levels, normalized to β-actin, was performed based on three independent experiments. Data were presented as Mean ± SD. *P < 0.05,, ***P < 0.001. ns no significance. **K**, **L** The indicated MDA-MB-231 and MDA-MB-468 cell lines were treated with or without MG132 (10 μM) for 12 h. The expression of DHODH and USP24 proteins was assessed by Western blotting. Quantification of protein levels, normalized to β-actin, was performed based on three independent experiments. Data were presented as Mean ± SD. *P < 0.05, **P < 0.01, ****P < 0.0001. ns no significance.

To elucidate the specific mechanism by which USP24 inhibits ferroptosis, we hypothesized that USP24 may influence the stability of key ferroptosis-related proteins. To test this, we examined the impact of *USP24* knockdown on several ferroptosis-associated proteins. Western blot assay revealed that *USP24* knockdown markedly downregulated DHODH protein levels in both MDA-MB-231 and MDA-MB-468 cells, while having relatively minor effects on other proteins, including GPX4, SLC7A11, FSP1, ACSL4, NRF2, ALOX12, ferritin, and p53 (Fig. 4A, B). The knockdown of USP24 does not affect GSS expression in MDA-MB-231 cells, but it downregulates GSS in MDA-MB-468 cells, indicating that the regulation of GSS is cell-type dependent (Fig. 4A, B). Interestingly, *USP24* knockdown had little impact on *DHODH* gene expression levels in either cell line (Supplementary Fig. 4A). Similarly, treatment with the USP24 inhibitor WP1130 reduced DHODH protein levels but did not affect its gene expression (Fig. 4C, D and Supplementary Fig. 4B). Given that WP1130 can also inhibit USP9X, we further investigated the effect of USP9X on DHODH protein expression. However, our results showed that knockdown of USP9X did not affect the protein levels of DHODH (Fig. 4E, F). These findings suggest that USP24 regulates DHODH protein levels through a post-transcriptional mechanism.

To further investigate whether USP24 affects DHODH at the post-translational level, we inhibited protein synthesis using cycloheximide (CHX). Despite blocking protein synthesis, *USP24* knockdown reduced DHODH protein levels in TNBC cells (Fig. 4G, H), supporting the hypothesis that USP24 regulates DHODH protein stability post-translationally.

Since intracellular protein degradation occurs predominantly through the ubiquitin–proteasome system (UPS) or autophagy [52], we sought to identify which pathway is involved in DHODH degradation. Treatment with the proteasome inhibitor MG132, but not the autophagy inhibitor chloroquine (CQ), led to a marked accumulation of DHODH protein (Fig. 4I, J), indicating that DHODH is primarily degraded via the UPS. Moreover, MG132 effectively blocked the degradation of DHODH in *USP24*-knockdown MDA-MB-231 and MDA-MB-468 cells (Fig. 4K, L), further confirming the role of the UPS in DHODH stability.

### USP24 inhibits ferroptosis by stabilizing DHODH protein

**Fig. 5:**
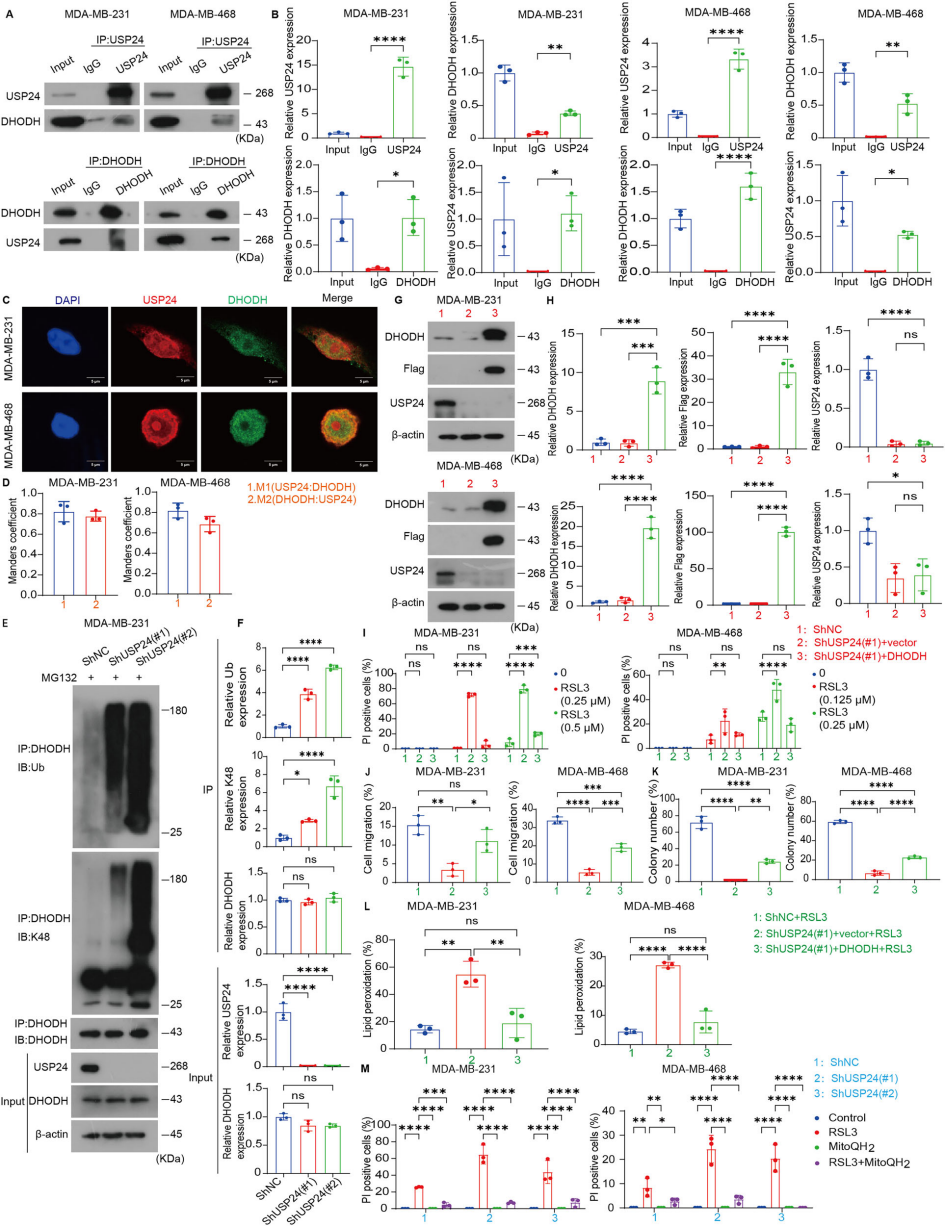
USP24 inhibits ferroptosis by increasing the protein stabilization of DHODH. **A**, **B** Endogenous USP24 and DHODH interactions were detected by coimmunoprecipitation using USP24 and DHODH antibodies, respectively, in MDA-MB-231 and MDA-MB-468 cells. Quantification of protein levels, normalized to β-actin, was performed based on three independent experiments. Data were presented as Mean ± SD. *P < 0.05, **P < 0.01, ****P < 0.0001. **C**, **D** The intracellular localization of USP24 (red) and DHODH (green) in MDA-MB-231 and MDA-MB-468 cells was examined by immunofluorescence staining using USP24 and DHODH antibodies and visualized by fluorescence microscopy. DAPI was used to stain nuclei. Scale bars: 5 μm. Immunofluorescence colocalization was quantified using the Manders’ coefficient. **E**, **F** The indicated MDA-MB-231 cell lines were pretreated with MG132 (10 μM) for 24 h, and then the extracts were immunoprecipitated with anti-DHODH antibodies and immunoblotted with anti-ubiquitin (Ub), anti-K48-linked ubiquitin, and anti-DHODH antibodies. Quantification of protein levels, normalized to β-actin, was performed based on three independent experiments. Data were presented as Mean ± SD. *P < 0.05, ****P < 0.0001. ns no significance. **G**, **H** MDA-MB-231 and MDA-MB-468 cells stably transfected with USP24 shRNA#1 or control shRNA were stably expressed with Flag-DHODH or control vector. USP24 and DHODH expression in cells was measured by Western blotting. Quantification of protein levels, normalized to β-actin, was performed based on three independent experiments. Data were presented as Mean ± SD. *P < 0.05, ***P < 0.001, ****P < 0.0001. ns no significance. **I** Cell death was assessed using propidium iodide (PI) staining in the indicated MDA-MB-231 and MDA-MB-468 cell lines treated with the indicated doses of RSL3 for 24 h. Quantification of PI-positive cells was shown. Mean ± SD, n = 3. **P < 0.01, ***P < 0.001, ****P < 0.0001. ns no significance. **J**, **K** Migration (**J**) and cloning formation (**K**) abilities of the indicated MDA-MB-231 and MDA-MB-468 cell lines treated with RSL3 (0.5 μM for MDA-MB-231 cells; 0.25 μM for MDA-MB-468 cells) for 6 h. Mean ± SD, n = 3. *P < 0.05, **P < 0.01, ***P < 0.001, ****P < 0.0001. ns no significance. **L** Lipid peroxidation of the indicated MDA-MB-231 and MDA-MB-468 cell lines treated with RSL3 (0.5 μM for MDA-MB-231 cells; 0.25 μM for MDA-MB-468 cells) for 6 h. Mean ± SD, n = 3. **P < 0.01, ****P < 0.0001. ns no significance. **M** Cell death was assessed using propidium iodide (PI) staining in the indicated MDA-MB-231 and MDA-MB-468 cell lines treated with RSL3 (0.5 μM for MDA-MB-231 cells; 0.25 μM for MDA-MB-468 cells) in the presence or absence of MitoQH_2_ (2.5 μM) for 24 h. Quantification of PI-positive cells was shown. Mean ± SD, n = 3. *P < 0.05, **P < 0.01, ***P < 0.001, ****P < 0.0001.

The primary function of DUBs is to remove ubiquitin from substrate proteins, thereby enhancing their stability. To verify whether USP24 directly interacts with DHODH, we performed co-immunoprecipitation experiments and found that USP24 co-immunoprecipitated with DHODH in MDA-MB-231 and MDA-MB-468 cells (Fig. 5A, B). Additionally, immunofluorescence analysis revealed that USP24 and DHODH co-localize primarily in the cytoplasm of MDA-MB-231 and MDA-MB-468 cells, supporting their functional association (Fig. 5C, D).

To further investigate the functional relationship, we examined the impact of *USP24* knockdown on DHODH ubiquitination. USP24 knockdown significantly increased both total and K48-linked ubiquitination of DHODH, suggesting that USP24 stabilizes DHODH by catalyzing its deubiquitination (Fig. 5E, F). These findings highlight the critical role of USP24 in maintaining DHODH protein stability through ubiquitination regulation.

Next, to determine whether USP24-mediated suppression of ferroptosis depends on DHODH, we overexpressed *DHODH* in *USP24*-knockdown cells. *DHODH* overexpression rescued the cell death induced by *USP24* knockdown in RSL3-treated MDA-MB-231 and MDA-MB-468 cells (Fig. 5G-I). Furthermore, cell migration and colony formation assays demonstrated that *DHODH* overexpression attenuated the inhibitory effects on cell migration and proliferation caused by *USP24* knockdown (Fig. 5J, K). Consistently, lipid peroxidation analysis using the Liperfluo probe showed that restoring DHODH expression reduced the elevated lipid peroxidation levels observed in *USP24*-knockdown cells (Fig. 5L).

Mechanistically, DHODH catalyzes the reduction of CoQ to its active form, CoQH₂, which is essential for mitigating lipid peroxidation and ferroptosis [27]. To further validate this mechanism, we supplemented *USP24*-knockdown cells with mitoQH₂, a mitochondria-targeted analog of CoQH₂. MitoQH₂ supplementation effectively protected against ferroptosis triggered by *USP24* knockdown in MDA-MB-231 and MDA-MB-468 cells, confirming the role of DHODH activity in ferroptosis suppression (Fig. 5M).

In summary, USP24 inhibits ferroptosis in TNBC cells by stabilizing DHODH protein levels through deubiquitination. This stabilization enhances DHODH activity, promoting CoQH₂ production and mitigating lipid peroxidation, thereby contributing to ferroptosis resistance in TNBC cells.

### USP24 inhibits RSL3-induced tumor suppression in TNBC cells in vivo

**Fig. 6:**
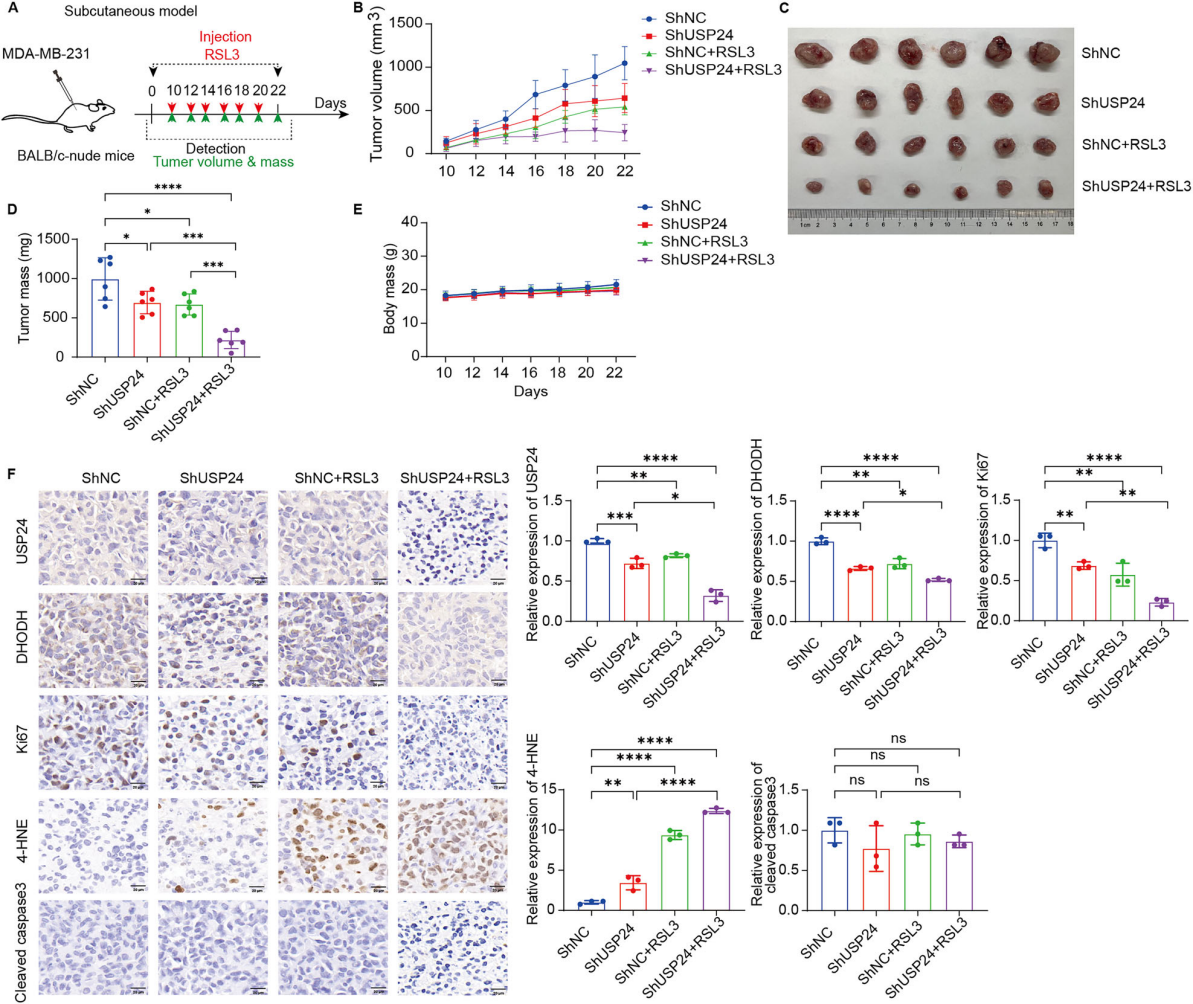
USP24 inhibits RSL3-induced tumor suppression in TNBC in vivo. **A** Schematic of the anticancer effect of RSL3 (2 mg/kg/2 days) in the indicated MDA-MB-231 tumor xenograft models. **B** The tumor volume of each group was calculated every 2 days. **C** The excised tumors were photographed on day 22. **D** The tumor weights of each group. Mean ± SD, n = 6. *P < 0.05, ***P < 0.001, ****P < 0.0001. **E** The mice body weight of each group was recorded every 2 days. **F** The expression of USP24, DHODH, Ki67, 4-HNE, and cleaved-caspase 3 were detected by immunohistochemical staining. Representative images and quantification in each group were shown (n = 3 samples). Scale bar, 20 μm. Mean ± SD, n = 3. *P < 0.05, **P < 0.01, ***P < 0.001, ****P < 0.0001. ns no significance.

To investigate the role of USP24 in ferroptosis in vivo, we established a subcutaneous xenograft model by injecting *USP24*-knockdown and control MDA-MB-231 cells into BALB/c nude mice (Fig. 6A). Once tumors formed, the mice were treated with the ferroptosis inducer RSL3. *USP24* knockdown enhanced RSL3-induced tumor suppression, as evidenced by reductions in tumor volume (Fig. 6B), tumor size (Fig. 6C), and tumor weight (Fig. 6D), without affecting the body weight of the mice (Fig. 6E).

Immunohistochemical analysis revealed a marked reduction in DHODH and Ki67 (a proliferation marker) in USP24-knockdown tumors, accompanied by elevated levels of 4-hydroxynonenal (4-HNE), a lipid peroxidation marker, but no changes in cleaved caspase-3, a apoptosis marker (Fig. 6F).

**Fig. 7:**
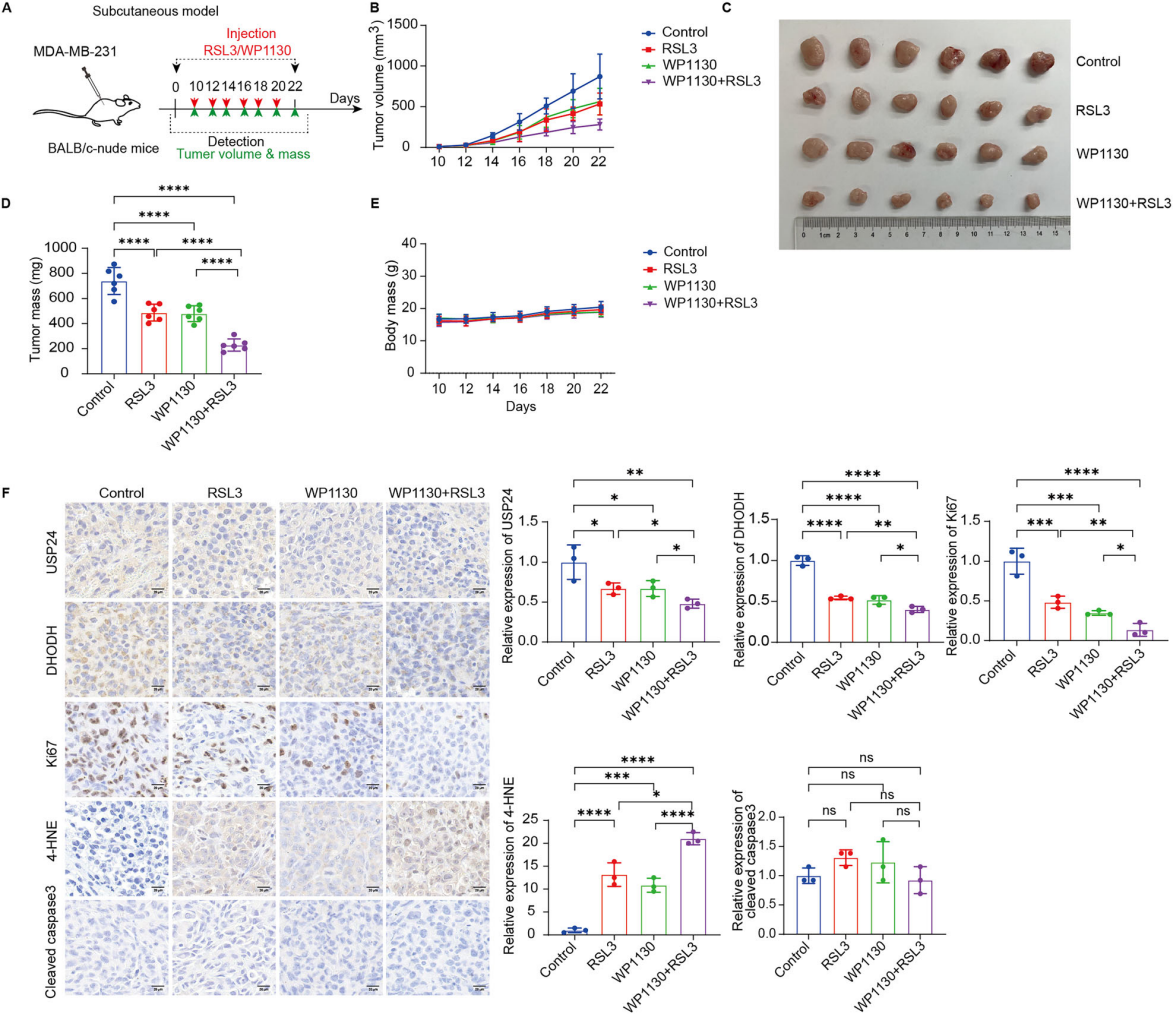
USP24 inhibitor promotes RSL3-induced tumor suppression in TNBC in vivo. **A** Schematic of the anticancer effect of RSL3 (2 mg/kg/2 days) with or without WP1130 (25 mg/kg/2 days) in the MDA-MB-231 tumor xenograft models. **B** The tumor volume of each group was calculated every 2 days. **C** The excised tumors were photographed on day 22. **D** The tumor weights of each group. Mean ± SD, n = 6. ****P < 0.0001. **E** The mice body weight of each group was recorded every 2 days. **F** The expression of USP24, DHODH, Ki67, 4-HNE, and cleaved-caspase 3 were detected by immunohistochemical staining. Representative images and quantification in each group were shown (n = 3 samples). Scale bar, 20 μm. Mean ± SD, n = 3. *P < 0.05, **P < 0.01, ***P < 0.001, ****P < 0.0001. ns no significance.

Similarly, pharmacological inhibition of USP24 with WP1130 significantly promoted RSL3-mediated tumor suppression and lipid peroxidation in TNBC cells (Fig. 7A–F). The combination of WP1130 and RSL3 showed enhanced anti-tumor effects without adverse effects on mouse body weight.

These findings demonstrate that USP24 inhibits RSL3-induced tumor suppression in vivo by stabilizing DHODH, thereby supporting tumor survival in TNBC.

## Discussion

**Fig. 8:**
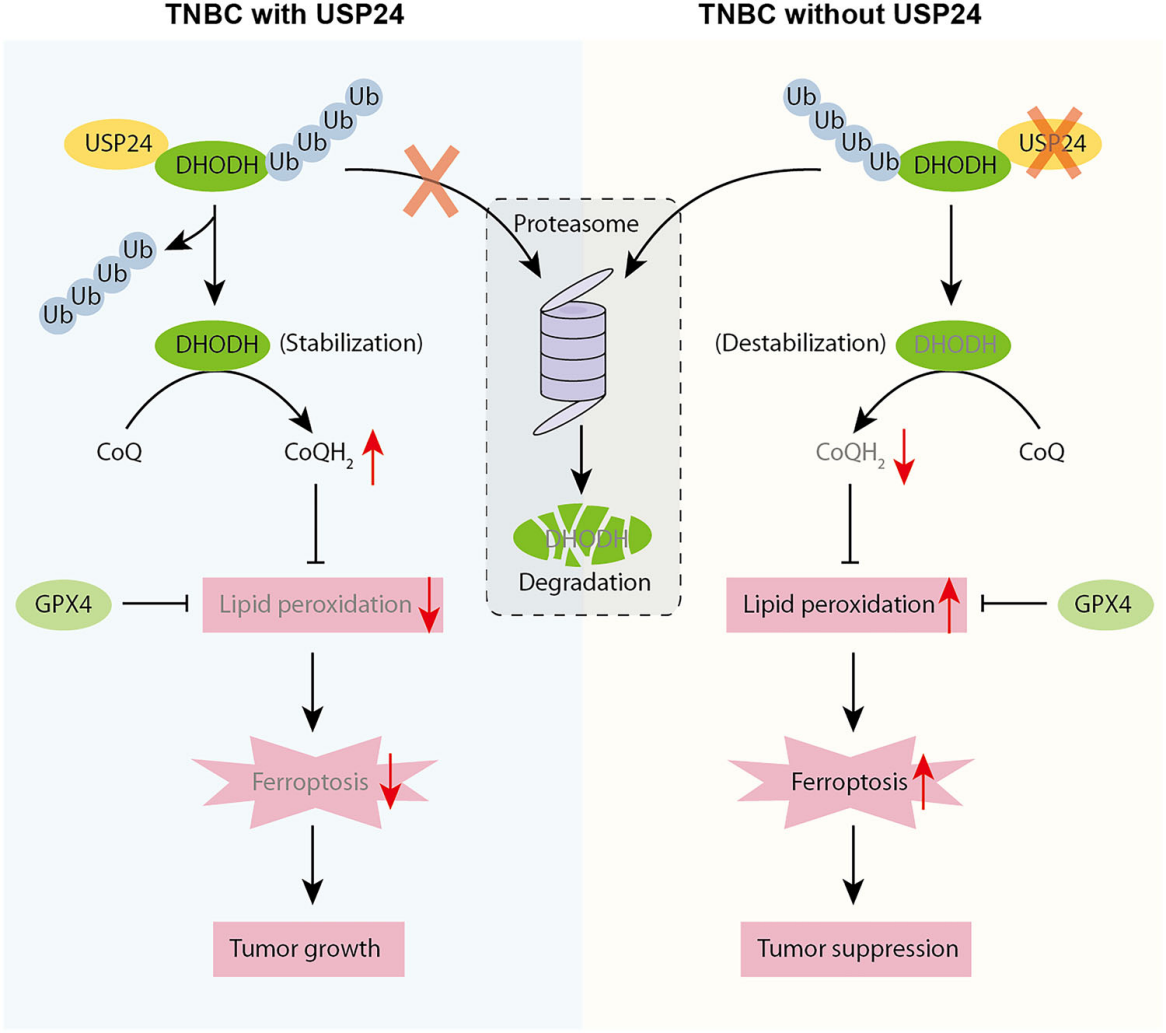
Working model for the function of USP24 in ferroptosis in TNBC. USP24 protects TNBC from ferroptosis by stabilizing DHODH in TNBC cells. USP24 blockade is synthetic lethal with GPX4 inhibitors, inducing ferroptosis through a DHODH-dependent mechanism.

TNBC presents significant challenges in clinical management, underscoring the urgent need for effective therapeutics targeting novel mechanisms of action [53]. DUBs counteract ubiquitination by removing ubiquitin from substrate proteins, a process that plays a pivotal role in breast cancer progression and therapeutic resistance [54]. Here, we identify USP24 as a key negative regulator of ferroptosis in TNBC. USP24 inhibits ferroptotic cell death through stabilizing DHODH protein in TNBC cells (Fig. 8).

The degradation pathways, including the UPS and autophagy, contribute to ferroptosis by removing key regulatory factors [55]. These pathways maintain cellular homeostasis by regulating the half-life of proteins involved in lipid metabolism, iron homeostasis, and oxidative stress—processes central to ferroptosis. Autophagy, in particular, enhances ferroptosis by selectively degrading proteins such as ferritin (ferritinophagy) to release free iron or GPX4 to impair the antioxidant system [56]. Understanding the context-dependent roles of degradation pathways in ferroptosis is crucial for developing novel anticancer therapies.

To the best of our knowledge, this study is the first to report a novel function of USP24 in inhibiting ferroptosis through the UPS pathway in TNBC. USP24, a member of the DUB family, is known to contribute to tumor progression by deubiquitinating and stabilizing substrate proteins [37, 38, 57]. It has been implicated as an oncogene in various cancers, including lung, bladder, and hepatocellular carcinomas [36–38, 58]. In contrast, USP24 has also been reported to act as a tumor suppressor in neuroblastoma [59]. Despite these findings, the specific role and molecular mechanisms of USP24 in TNBC remain largely unexplored. In this study, we demonstrate that USP24 is downregulated in response to GPX4 inhibitors, including RSL3, ML162, ML210, and FIN56. Functional shRNA experiments further confirm the role of USP24 in resisting ferroptosis in TNBC cells. Moreover, we identify WP1130, a known USP24 inhibitor, as a potential therapeutic agent for TNBC by enhancing ferroptosis. In addition, our findings are consistent with a previous study showing that pharmacological inhibition of USP24 promotes ferroptotic cell death in drug-resistant lung and brain cancer cells [44]. Intriguingly, USP24 uniquely promotes ferroptosis in hepatocellular carcinoma by removing ubiquitination from Beclin1, a core autophagy regulator [50]. Given the widespread expression of USP24, future research should comprehensively investigate the differences in USP24 substrates and ferroptosis sensitivity across various tumor types.

We examined USP24 expression in breast cancer (including TNBC) versus normal tissues using publicly available gene and protein databases. However, the results revealed no significant difference in USP24 protein levels between tumor and normal tissues, despite the lower USP24 gene expression in breast cancer compared to normal tissue (Supplementary Fig. 5A-F). In addition, breast cancer patients with elevated USP24 expression appear to have improved long-term survival outcomes (Supplementary Fig. 5G). However, we were unable to obtain relevant TNBC data, so the relationship between USP24 expression and TNBC survival outcomes remains unclear. These findings suggest that USP24 may not serve as a tumor-specific marker for TNBC and might play a limited role in tumorigenesis of TNBC. This uniform expression pattern could complicate the development of therapies designed to specifically target this protein. Furthermore, given the involvement of additional signaling pathways within tumors, it remains unclear whether USP24 intervention will yield differential responses in normal versus tumor tissues. Nonetheless, one promising strategy to overcome these challenges is to combine a ferroptosis inhibitor, which selectively downregulates USP24 in TNBC, with direct USP24 targeting. This combinatorial approach may enhance therapeutic efficacy while mitigating adverse side effects, ultimately paving the way for more effective treatment options.

Ferroptosis is closely associated with cellular GSH homeostasis, mediated by SLC7A11-dependent cystine uptake and GPX4 activity [60]. Compounds targeting this pathway, such as erastin, induce ferroptosis by depleting GSH and triggering endoplasmic reticulum (ER) stress [8, 61], while GPX4 inhibitors such as RSL3 irreversibly inactivate GPX4 by covalently binding to its active-site selenocysteine [46]. Our results reveal that GPX4 inhibitors significantly downregulate USP24 expression, potentially through an unidentified gene transcription mechanism. In contrast, erastin, an SLC7A11 inhibitor, exerts minimal effects on USP24 protein and gene expression, highlighting distinct regulatory pathways governing USP24 in response to different ferroptosis inducers. While the exact pathways remain unclear, we hypothesize that alternative metabolic pathways, such as the transsulfuration pathway [62], may compensate for ferroptotic effects induced by SLC7A11 inhibition. Further studies are needed to elucidate the role of these pathways in USP24-mediated ferroptosis regulation.

DHODH is an iron-containing, flavin-dependent enzyme localized to the inner mitochondrial membrane, playing a critical role in pyrimidine biosynthesis. Recently, it has garnered significant attention for its emerging role in inhibiting ferroptosis, although this function appears to be tumor type-dependent [63]. DHODH stability is tightly controlled by post-translational modifications, particularly ubiquitination. For example, DHODH is a downstream regulator of PRR11, which maintaining DHODH protein stability by inhibiting the binding between E3 ubiquitin ligase HERC4 and DHODH in glioblastoma [64]. Moreover, the lysyl-oxidase (LOX) family protein LOXL3 attenuates the ubiquitination of DHODH, in turn inducing the accumulation of DHODH proteins in liver cancer [65]. In this study, we identify USP24 as a DUB that directly removes ubiquitination from DHODH, stabilizing its expression in TNBC cells. While DHODH is a known mitochondrial protein, future experiments using mitochondrial markers will be necessary to further validate the interaction and potential colocalization of DHODH and USP24. Regardless, this finding highlights a novel regulatory mechanism of DHODH in ferroptosis and implicates USP24 as a critical mediator of DHODH-dependent survival in TNBC.

In summary, we establish USP24 as a critical regulator of ferroptosis in TNBC through its deubiquitination and stabilization of DHODH. Our findings provide new insights into the interplay between ubiquitination and ferroptosis, suggesting that USP24 could serve as a promising therapeutic target. Moreover, the combined use of ferroptosis inducers and USP24 inhibitors offers a potential strategy for improving therapeutic outcomes in TNBC by modulating DHODH stabilization.

## Supplementary information

Supplementary Figures

Uncropped western blot images

## Data Availability

The data that support the findings of this study are available from the corresponding author upon reasonable request.
