# Supplementary material for: The deubiquitinase USP24 suppresses ferroptosis in triple-negative breast cancer by stabilizing DHODH protein
Source: Cell Death Dis. 2025 Jul 26;16(1):564. doi: 10.1038/s41419-025-07895-4 (PMC12297596; doi:10.1038/s41419-025-07895-4)
Supplement: Supplementary file 1 — Supplementary Figures [file 41419_2025_7895_MOESM1_ESM.docx]

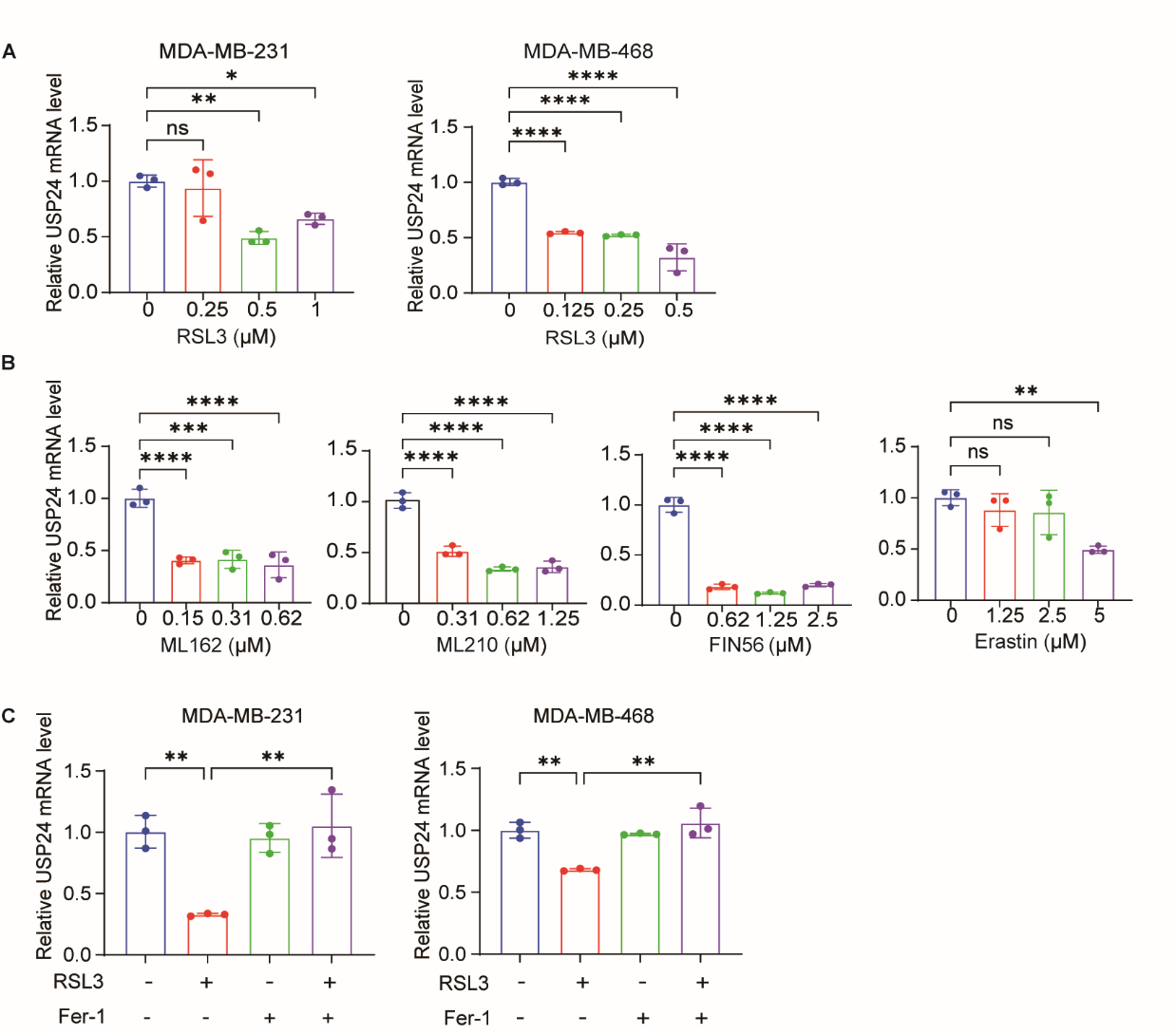


**Supplementary Figure 1. USP24 is downregulated during ferroptosis induced by GPX4 inhibitors. (A)** MDA-MB-231 and MDA-MB-468 cells were treated with RSL3 for 12 hours, and then USP24 mRNA levels were analyzed with qPCR. Mean ± SD, n=3. **P*<0.05, ***P*<0.01, *****P*<0.0001. ns, no significance. **(B)** MDA-MB-231 cells were treated with the indicated concentrations of ferroptosis inhibitors including ML162, ML210, FIN56, and erastin for 12 hours, and then USP24 mRNA levels were analyzed with western blotting and qPCR, respectively. Mean ± SD, n=3. ***P*<0.01, ****P*<0.001, *****P*<0.0001. ns, no significance. **(C)** qPCR was used to test the effects of ferroptosis inhibitor ferrostatin-1 (Fer-1, 2 μM) on RSL3 (0.5 μM for MDA-MB-231 cells; 0.25 μM for MDA-MB-468 cells, 12 hours)-induced USP24 downregulation. Mean ± SD, n=3. ***P*<0.01.


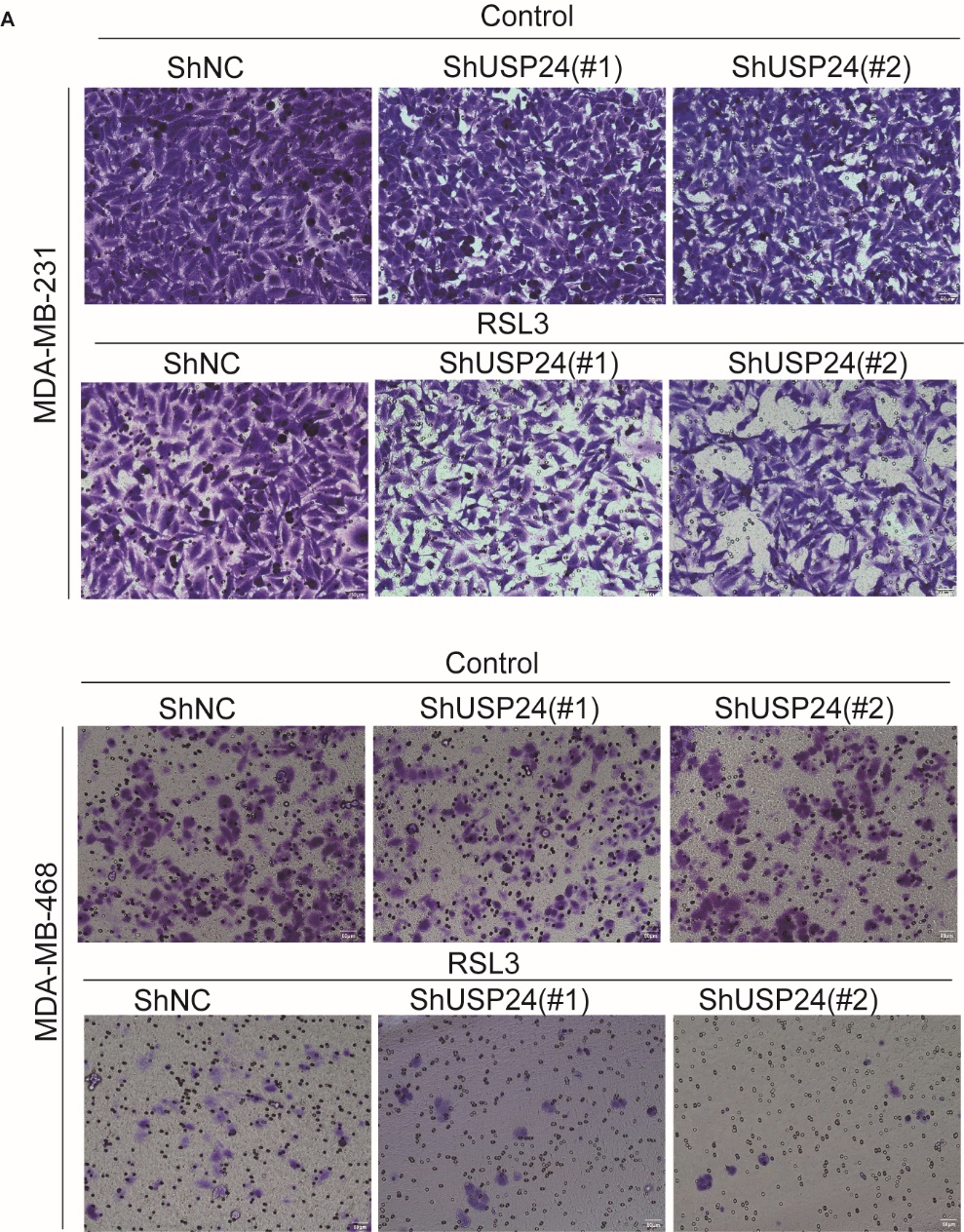


**Supplementary Figure 2. Knockdown of USP24 promotes ferroptosis in TNBC cells. (A)** Migration abilities of the indicated MDA-MB-231 and MDA-MB-468 cell lines treated with RSL3 (0.5 μM for MDA-MB-231 cells; 0.25 μM for MDA-MB-468 cells) for 6 hours.


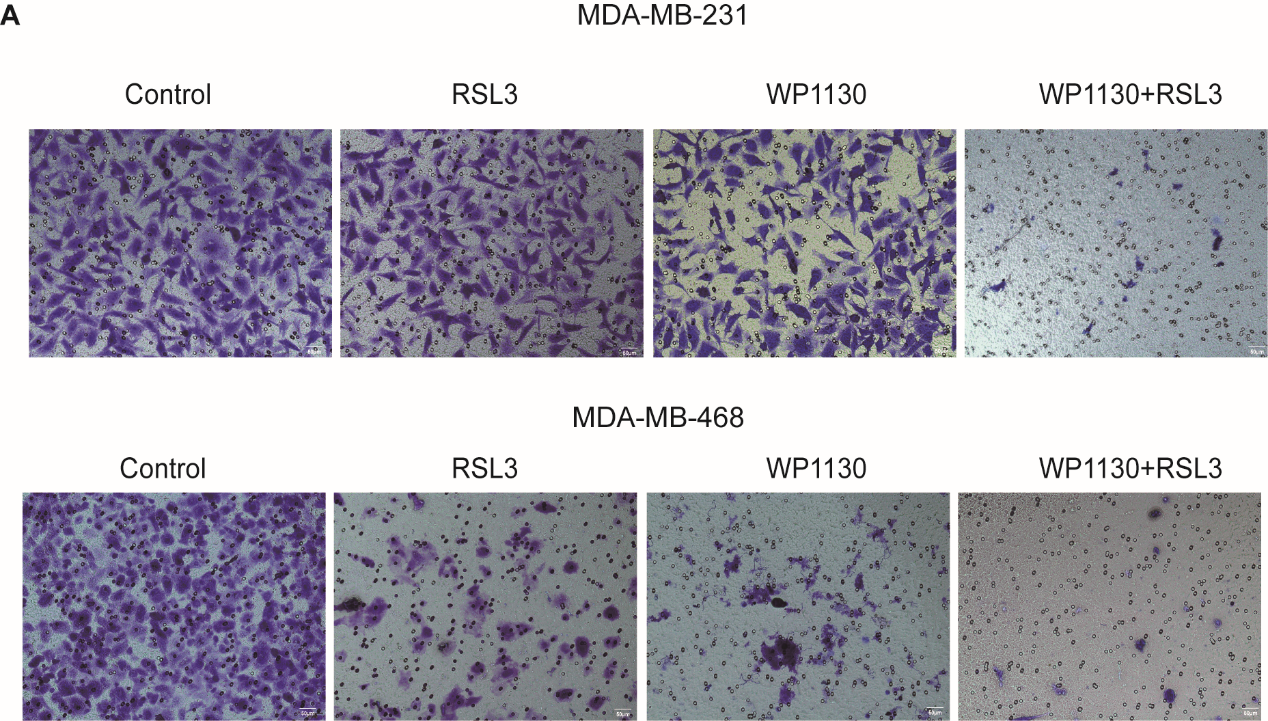


**Supplementary Figure 3. USP24 inhibitor exhibits a synergistic effect with ferroptosis inducer in TNBC cells. (A)** Migration abilities of MDA-MB-231 cells and MDA-MB-468 cells treated with RSL3 (0.5 μM for MDA-MB-231 cells; 0.25 μM for MDA-MB-468 cells) in the presence or absence of WP1130 (0.5 μM) for 6 hours.


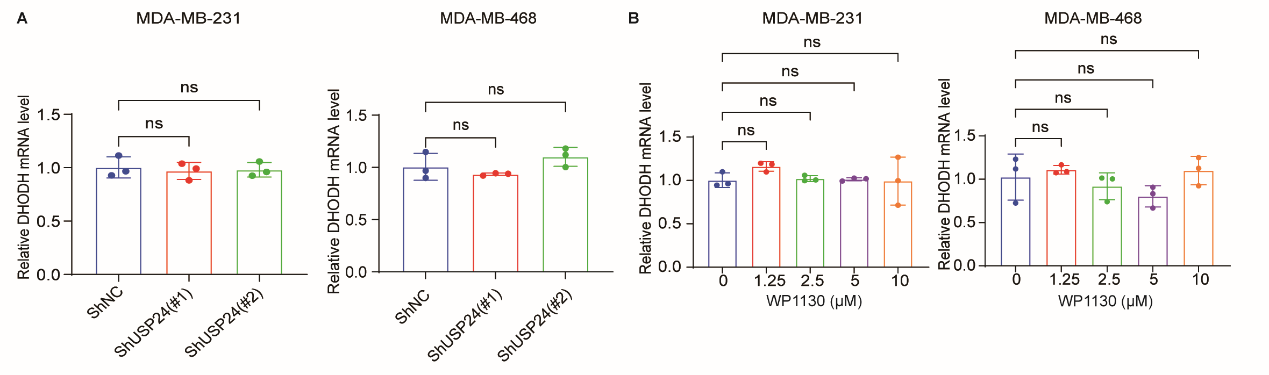


**Supplementary Figure 4.** **DHODH is a substrate of USP24 in TNBC cells. (A)** qPCR analysis of DHODH gene expression in the indicated MDA-MB-231 and MDA-MB-468 cell lines. **(B)** qPCR analysis of DHODH gene expression in MDA-MB-231 and MDA-MB-468 cells treated with the indicated doses of WP1130 for 24 hours. Mean ± SD, n=3. ns, no significance.


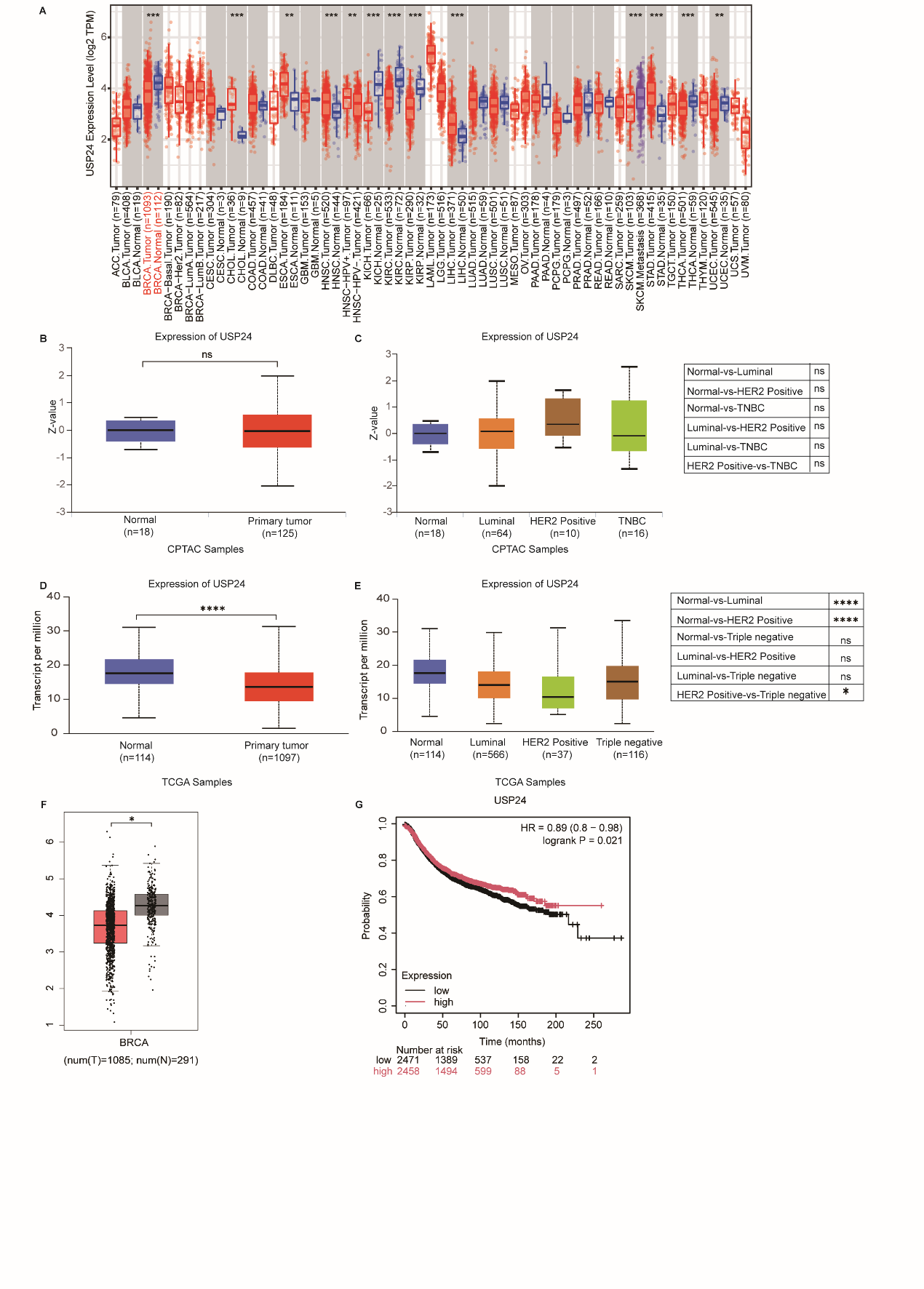


**Supplementary Figure 5. The predictive and prognostic role of USP24 in breast cancer and TNBC.**

(A) TIMER 2.0 database was used for pan‐cancer analysis of USP24. Mean ± SD. ****p<0.001. (B) Boxplot showing the relative protein expression of USP24 in normal and breast cancer samples using the CPTAC web resource. Mean ± SD, ns, no significance. (C) Box plot showing relative protein expression of USP24 in normal and major subclass of breast cancer using the CPTAC web resource. Mean ± SD, ns, no significance. (D) Boxplot showing the relative mRNA levels of USP24 in normal and breast cancer samples using the TCGA web resource. Mean ± SD. ****p<0.0001. (E) Box plot showing relative mRNA levels of USP24 in normal and major subclass of breast cancer using the TCGA web resource. Mean ± SD.*p<0.05, ****p<0.0001, ns, no significance. (F) Boxplot showing the relative mRNA levels of USP24 in normal and breast cancer samples using the GEPIA web resource. Mean ± SD. **p<0.01. (G) Association between USP24 expression and breast cancer patient survival. Data analyses were performed using Kaplan‒Meier Plotter.
