# Supplementary material for: The deubiquitinase USP24 suppresses ferroptosis in triple-negative breast cancer by stabilizing DHODH protein
Source: Cell Death Dis. 2025 Jul 26;16(1):564. doi: 10.1038/s41419-025-07895-4 (PMC12297596; doi:10.1038/s41419-025-07895-4)

Original image:Fig.1A

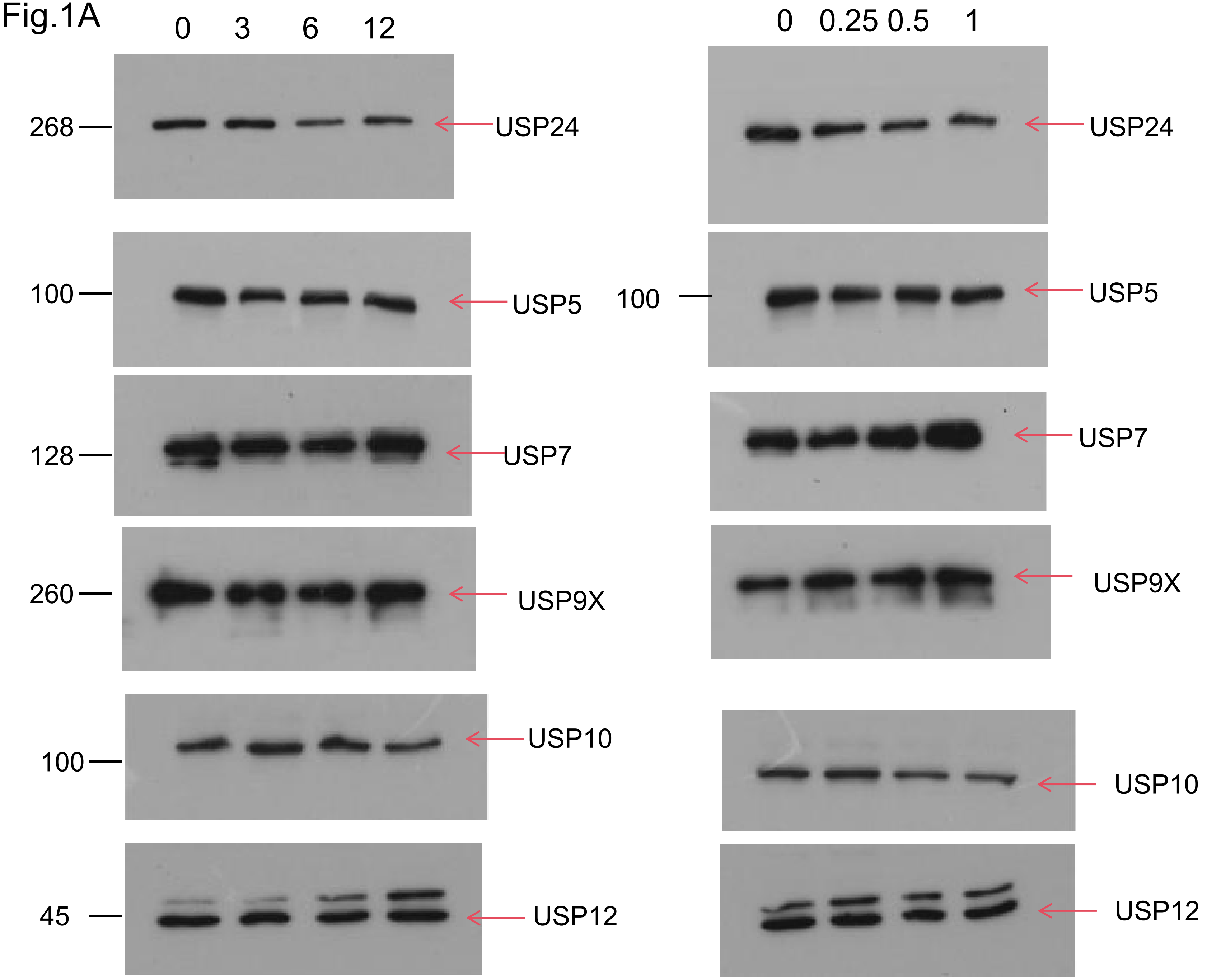

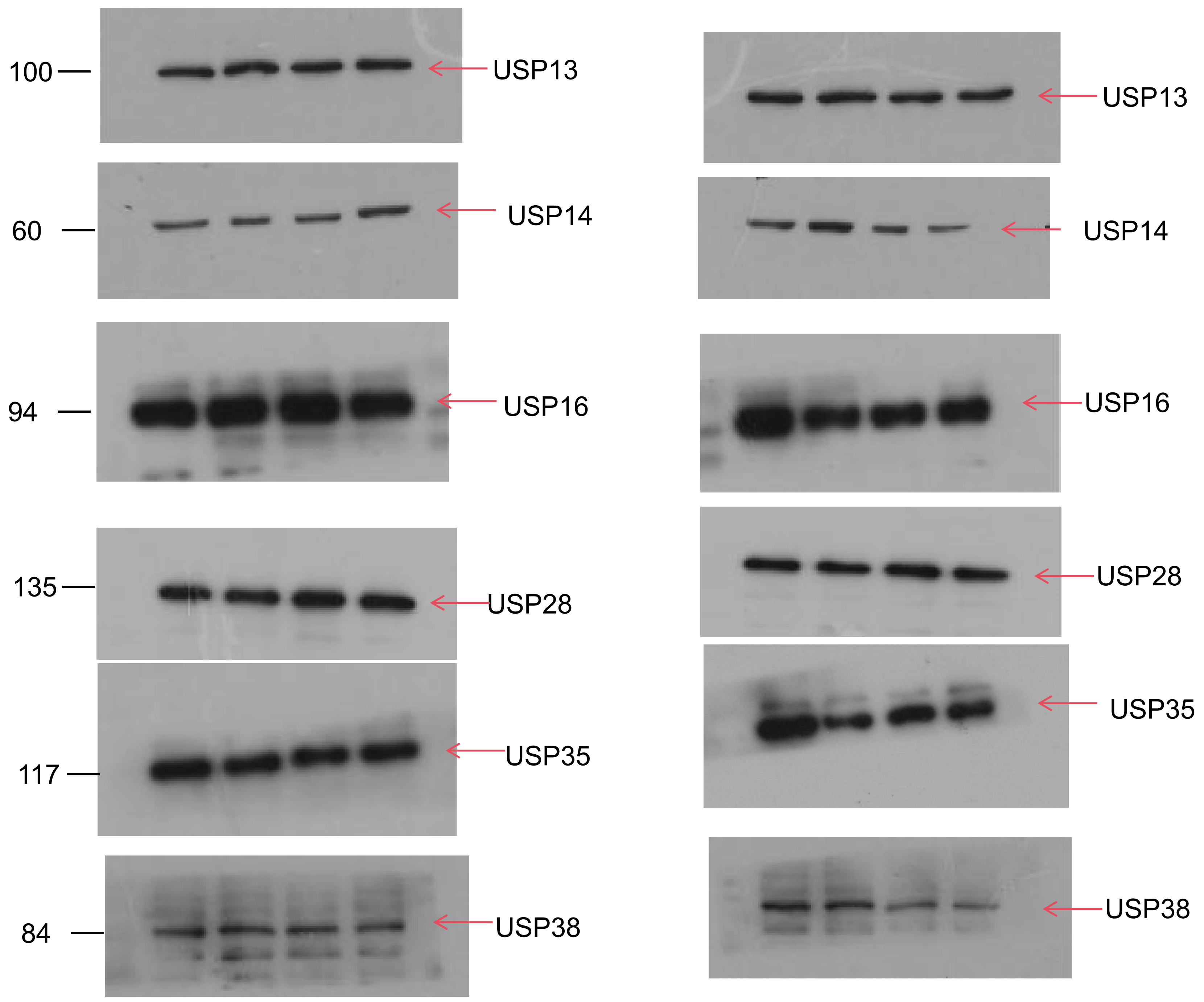
100
USP13
60
USP14
94
USP16
135
USP28
117
USP35
84
USP38
USP13
USP14
USP16
USP28
USP35
USP38

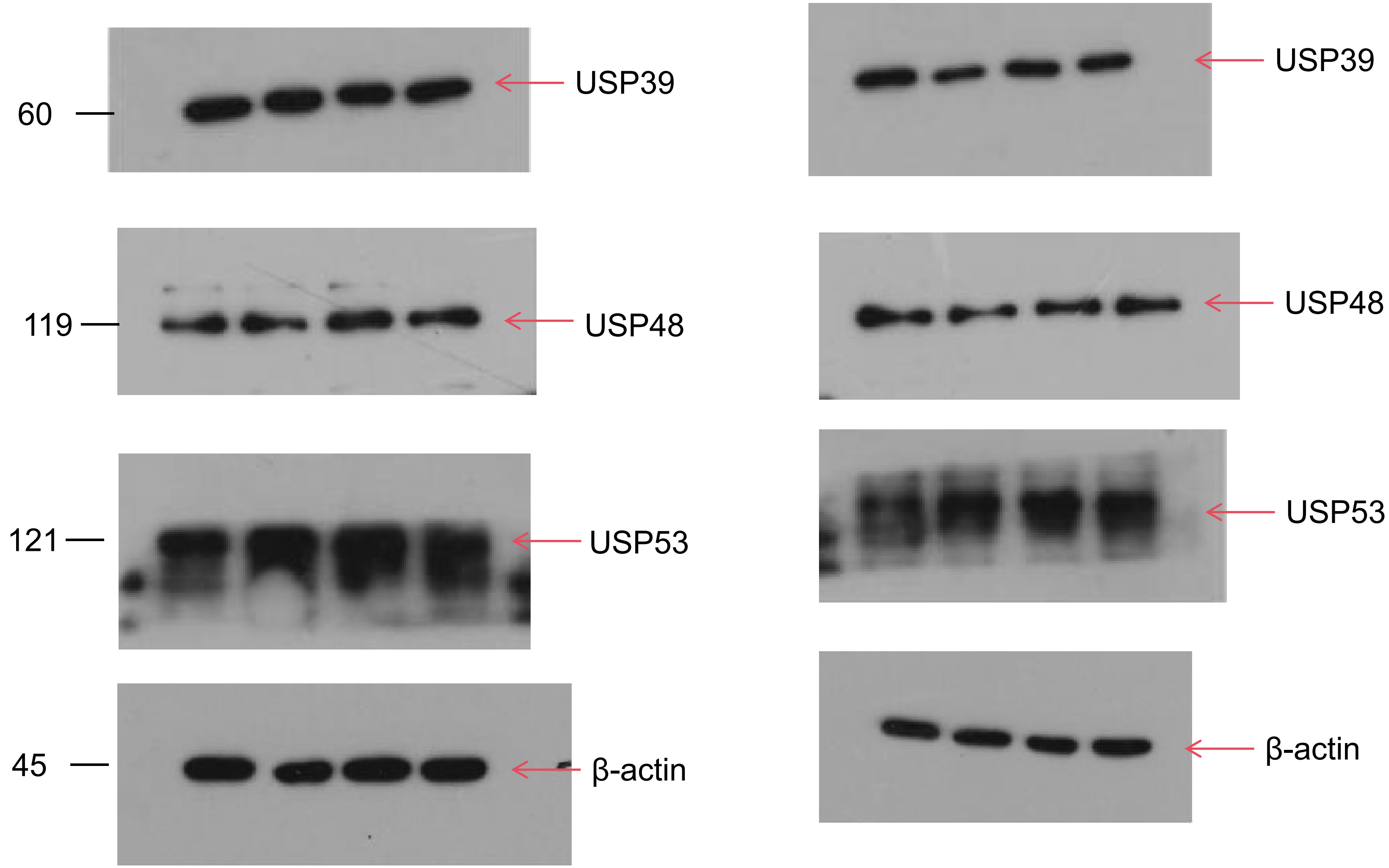

60
USP39
119
USP48
121
USP53
45
β-actin
USP39
USP48
USP53
β-actin

Original image: Fig.1C

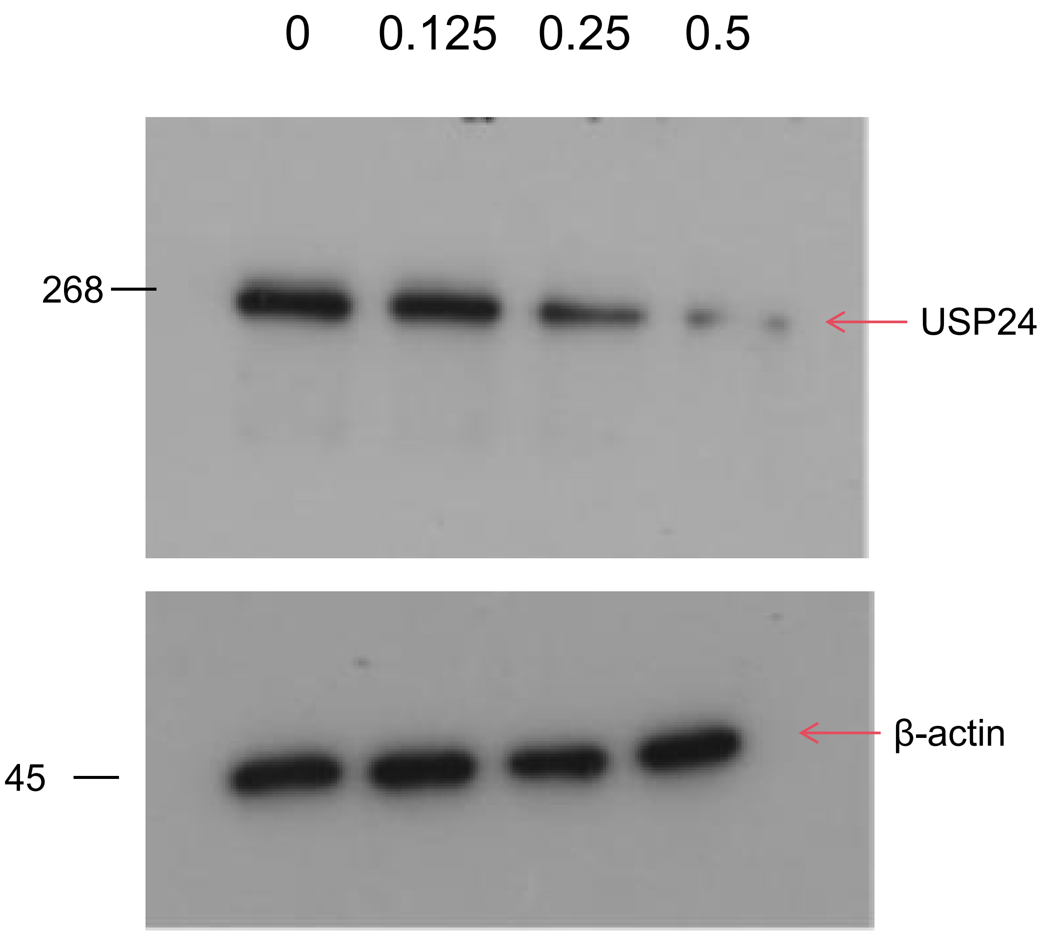

Original image: Fig.1E

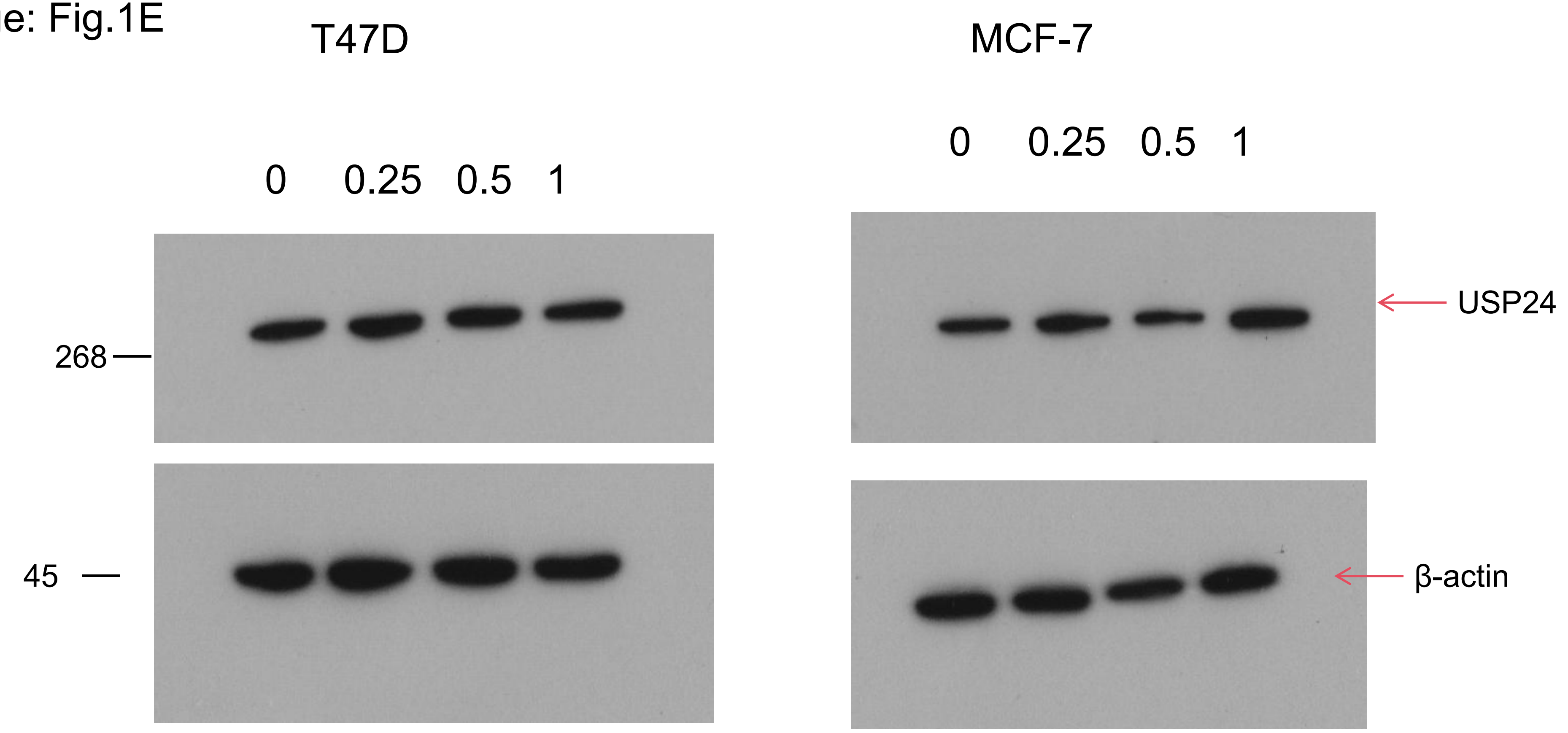

Original image: Fig.1G

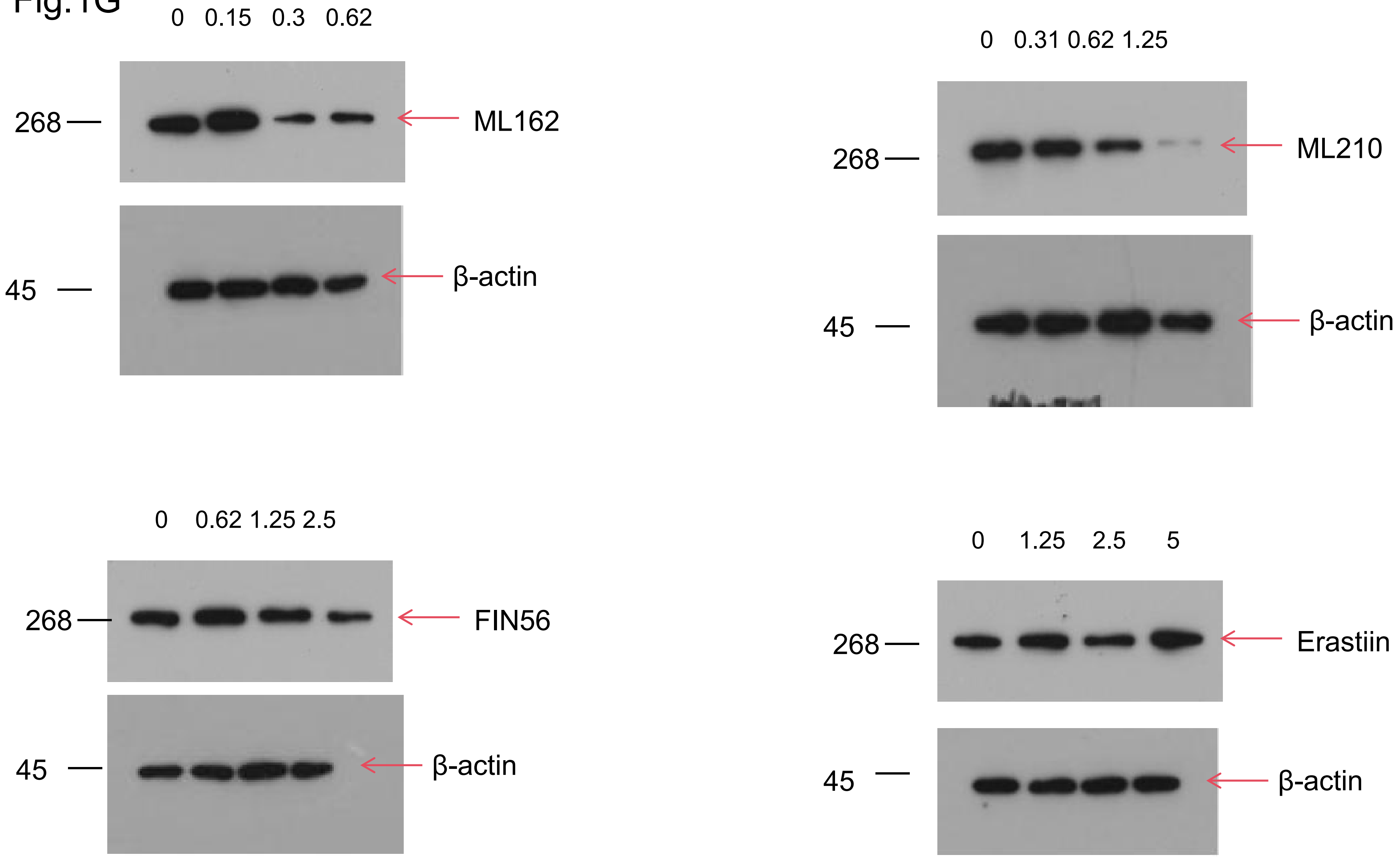

Original image:Fig.1I

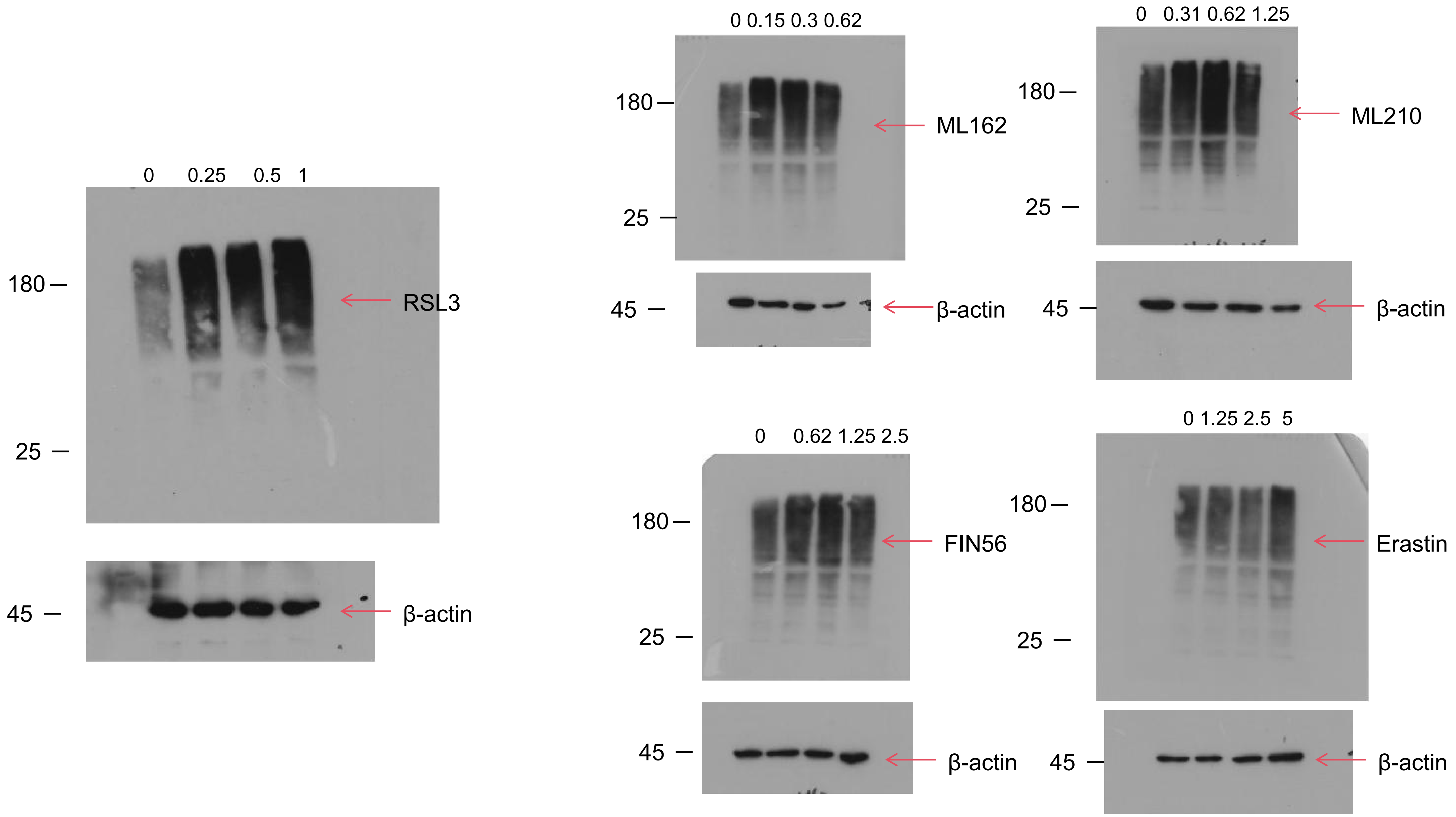

Original image: Fig.1K

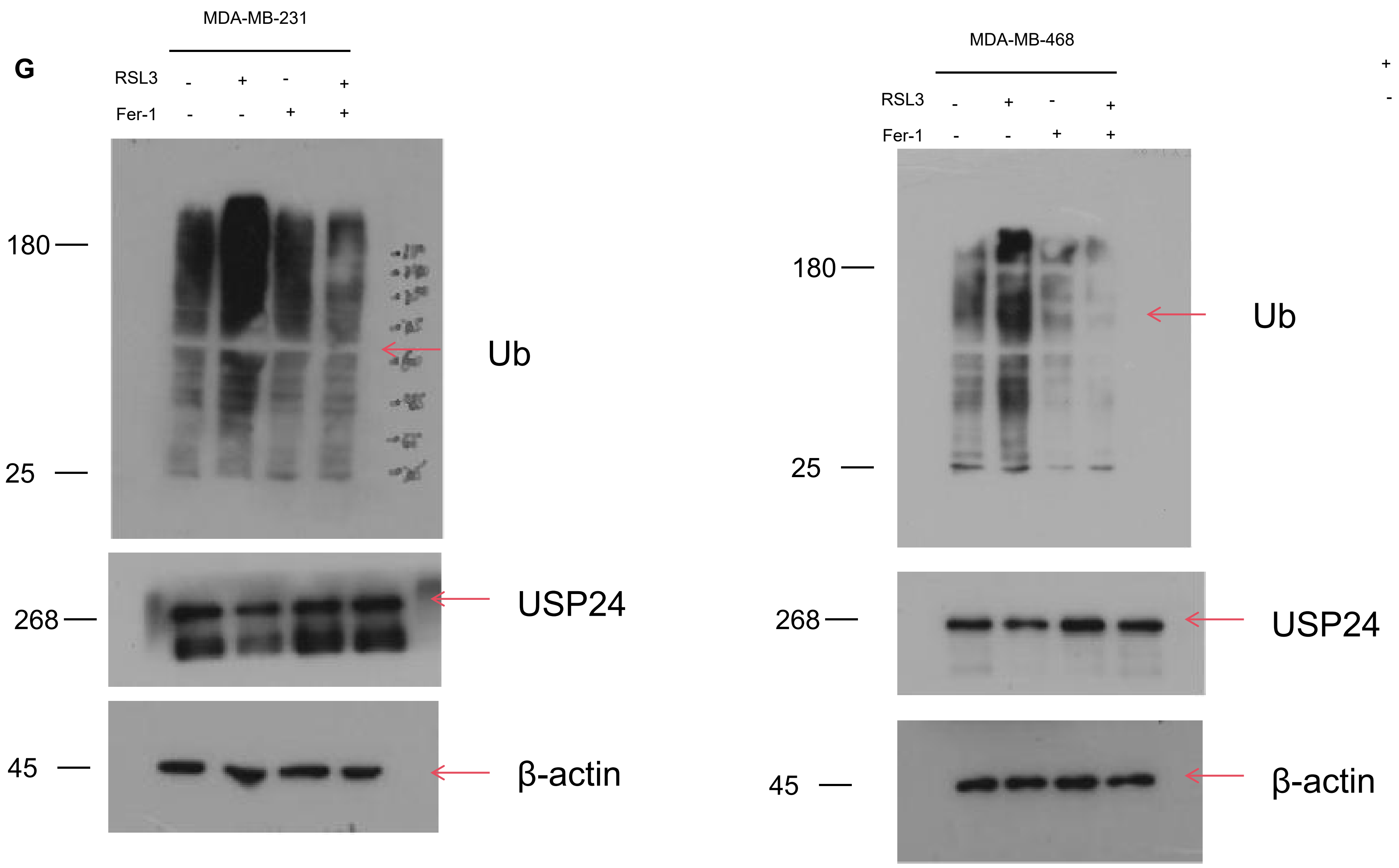

Original image:Fig.2A

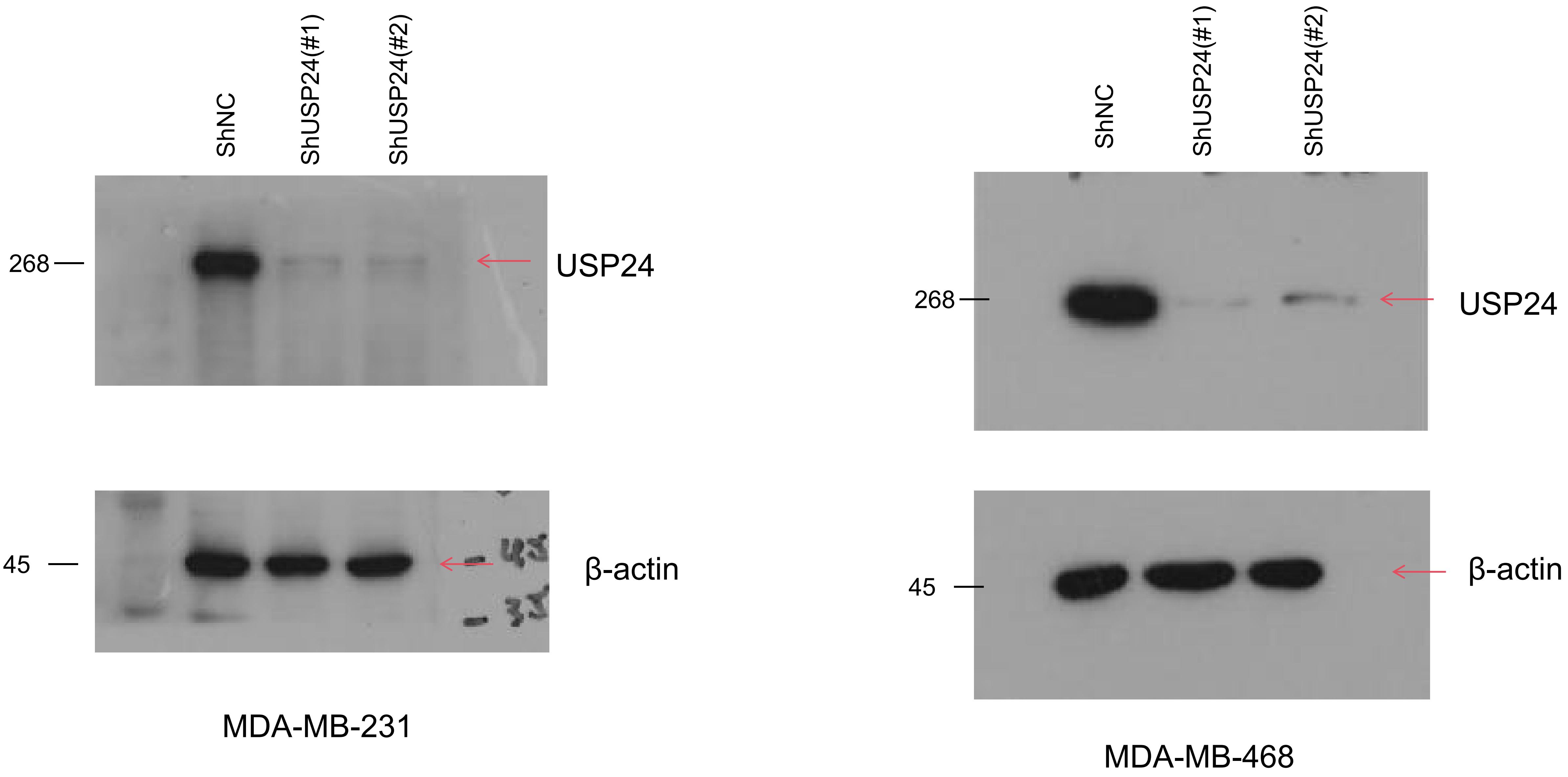

Original image:Fig.4A

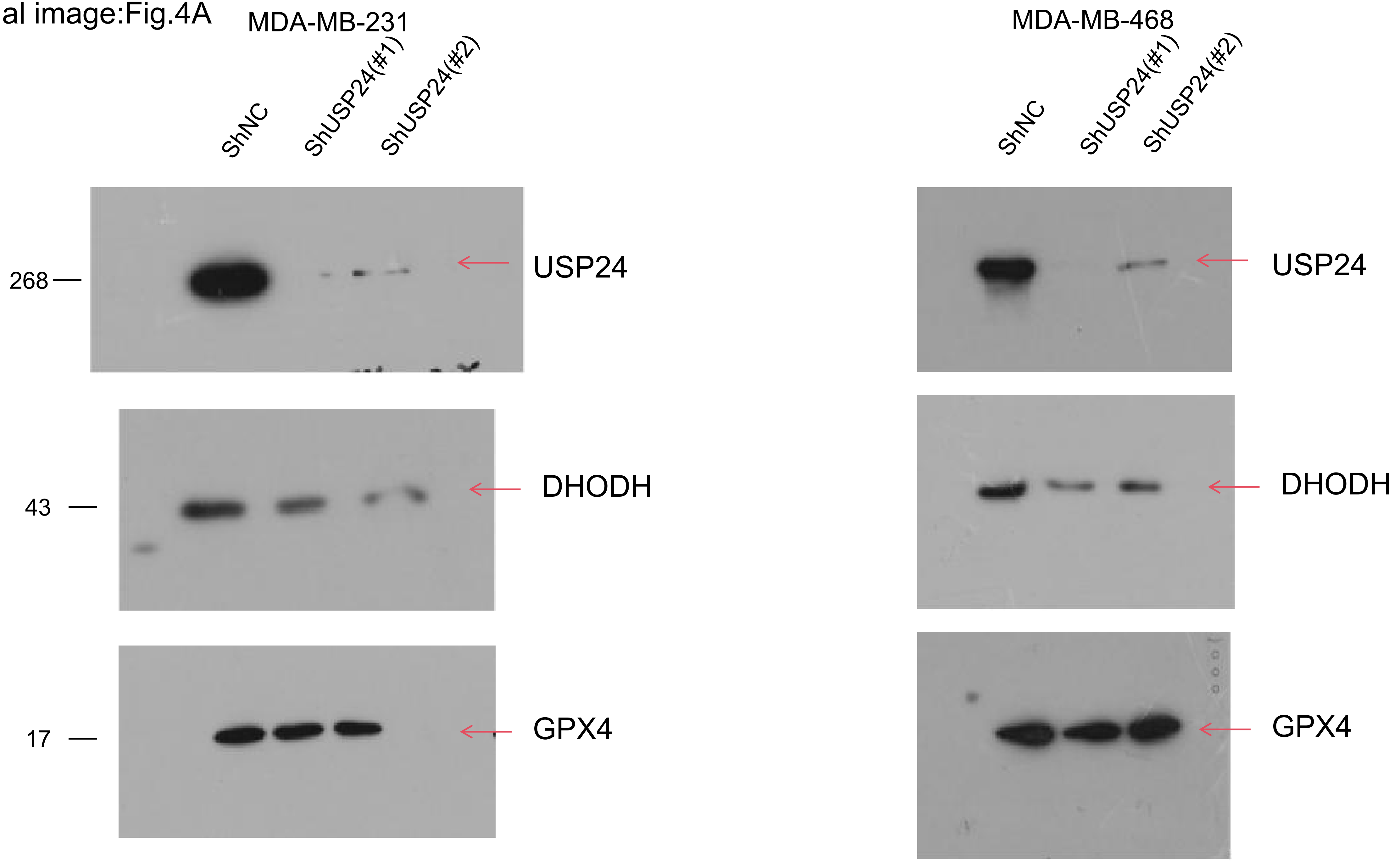

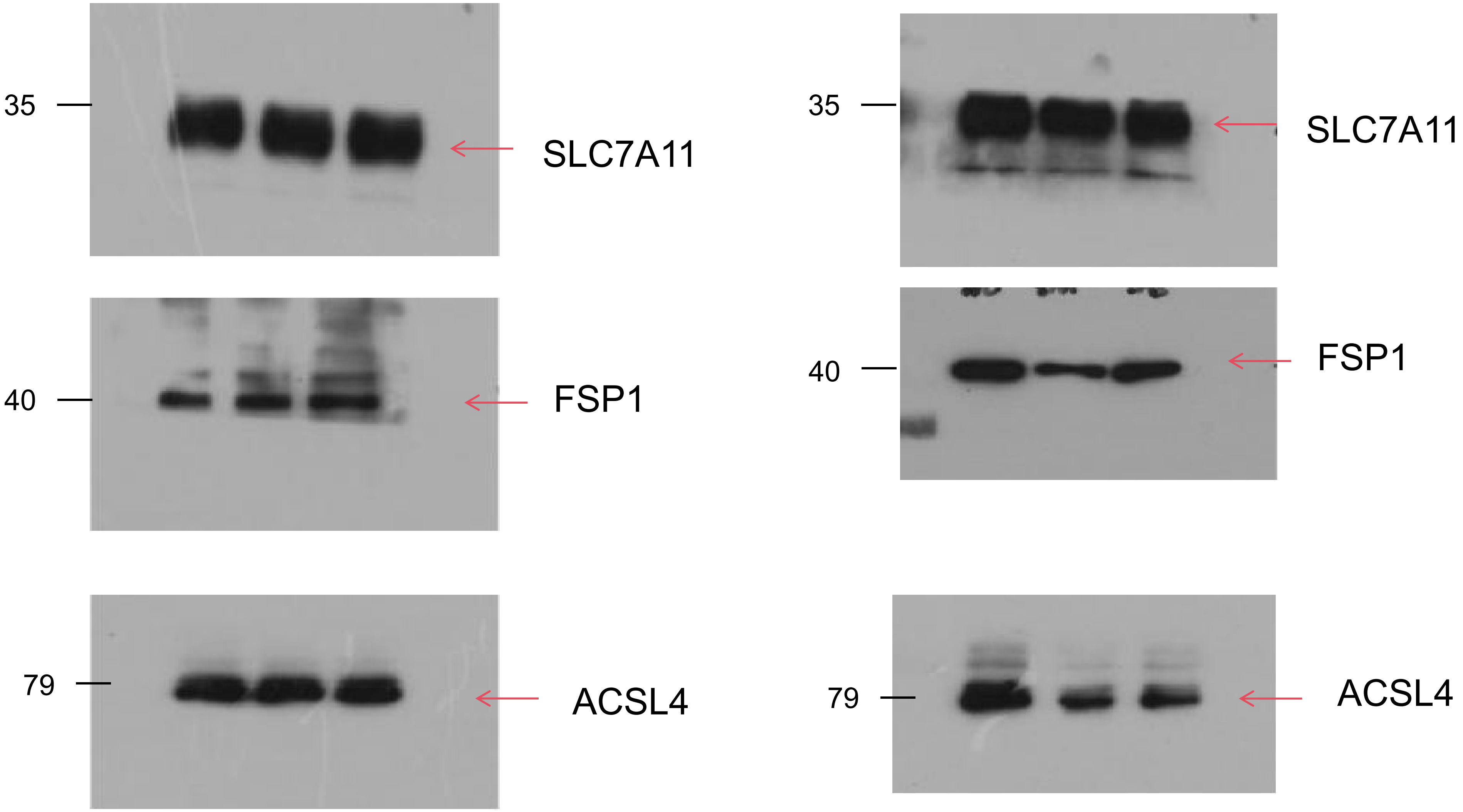
35
SLC7A11
40
FSP1
79
ACSL4
35
SLC7A11
40
FSP1
79
ACSL4

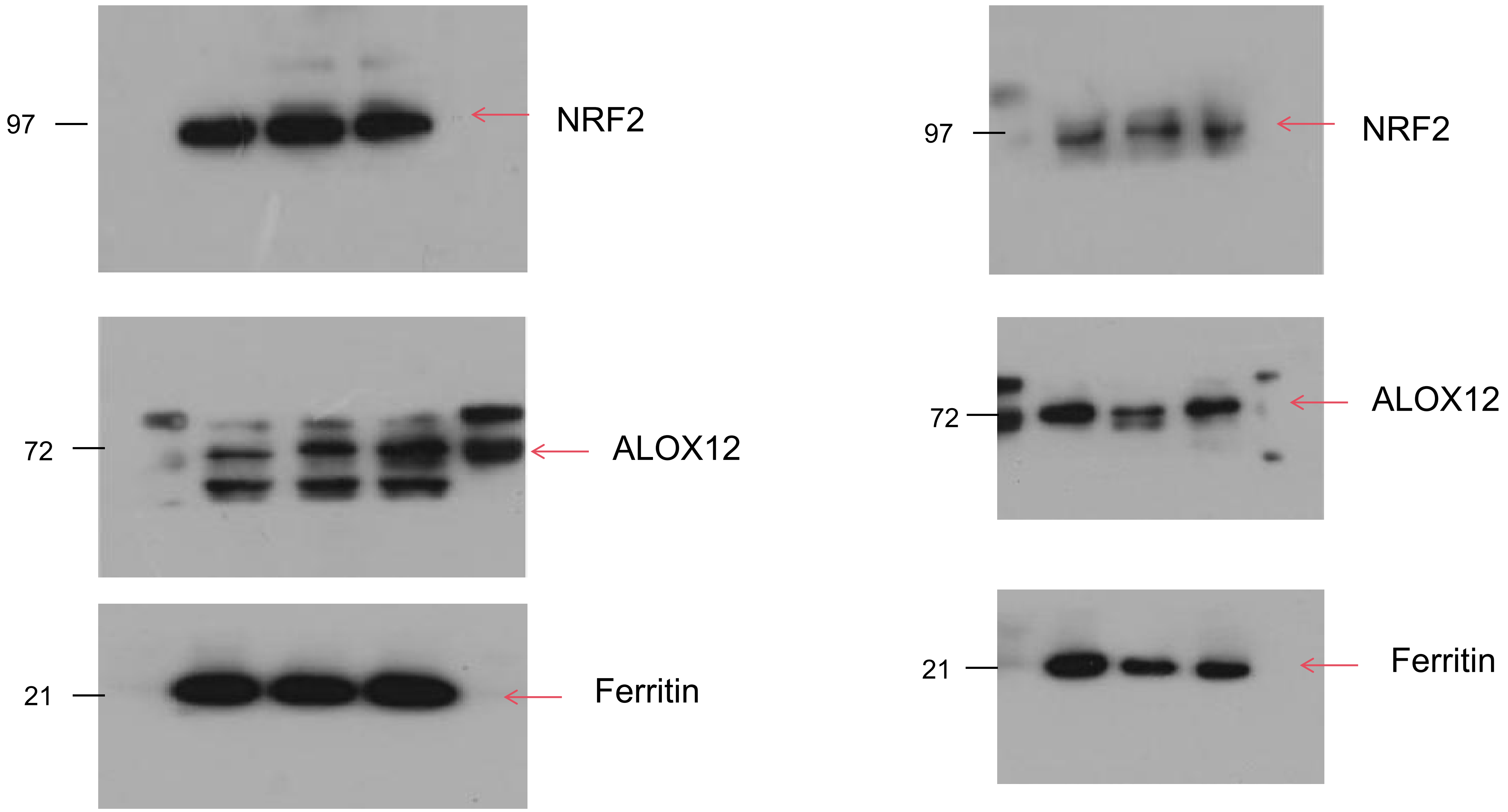
97
NRF2
72
ALOX12
21
Ferritin
97
NRF2
72
ALOX12
21
Ferritin

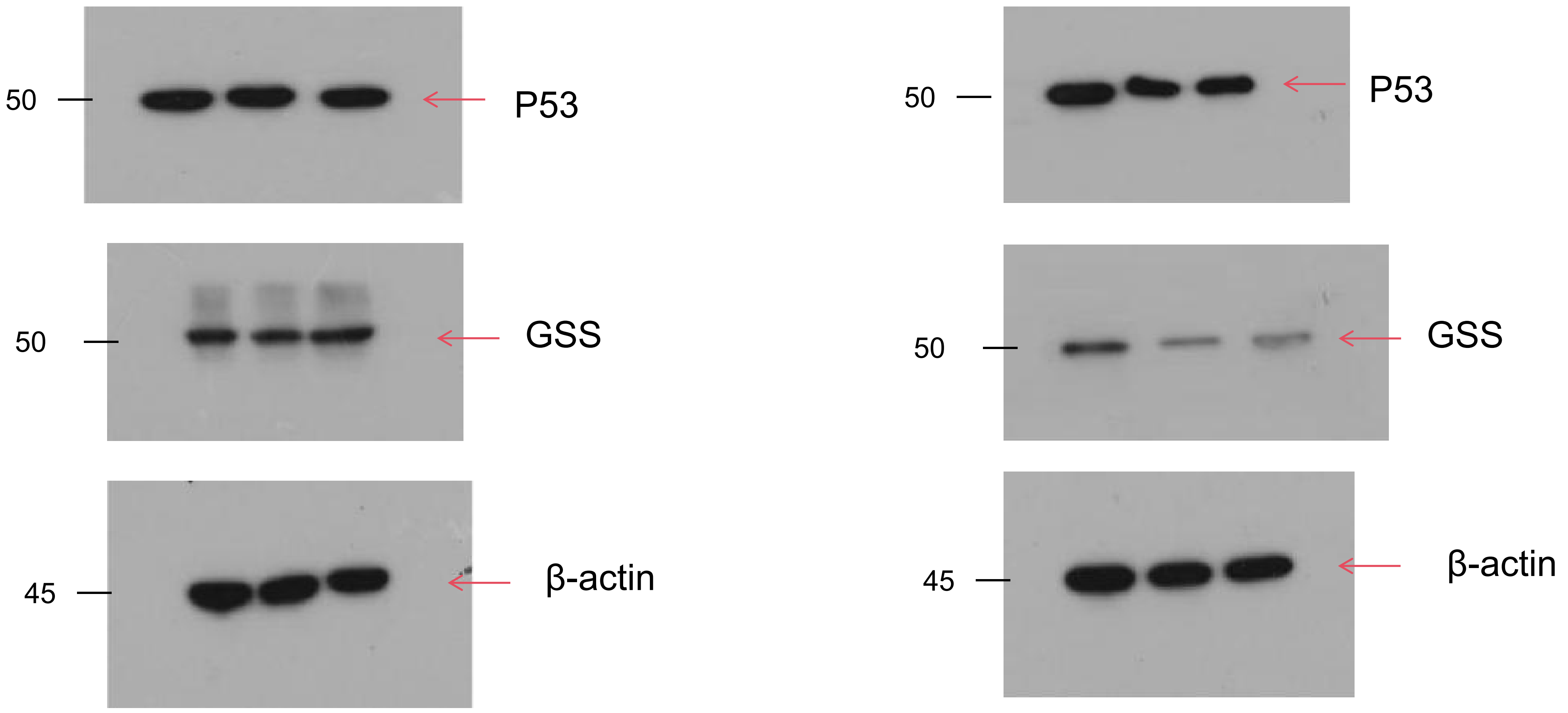

50
P53
50
GSS
45
β-actin
50
P53
50
GSS
45
β-actin

Original image:Fig.4C

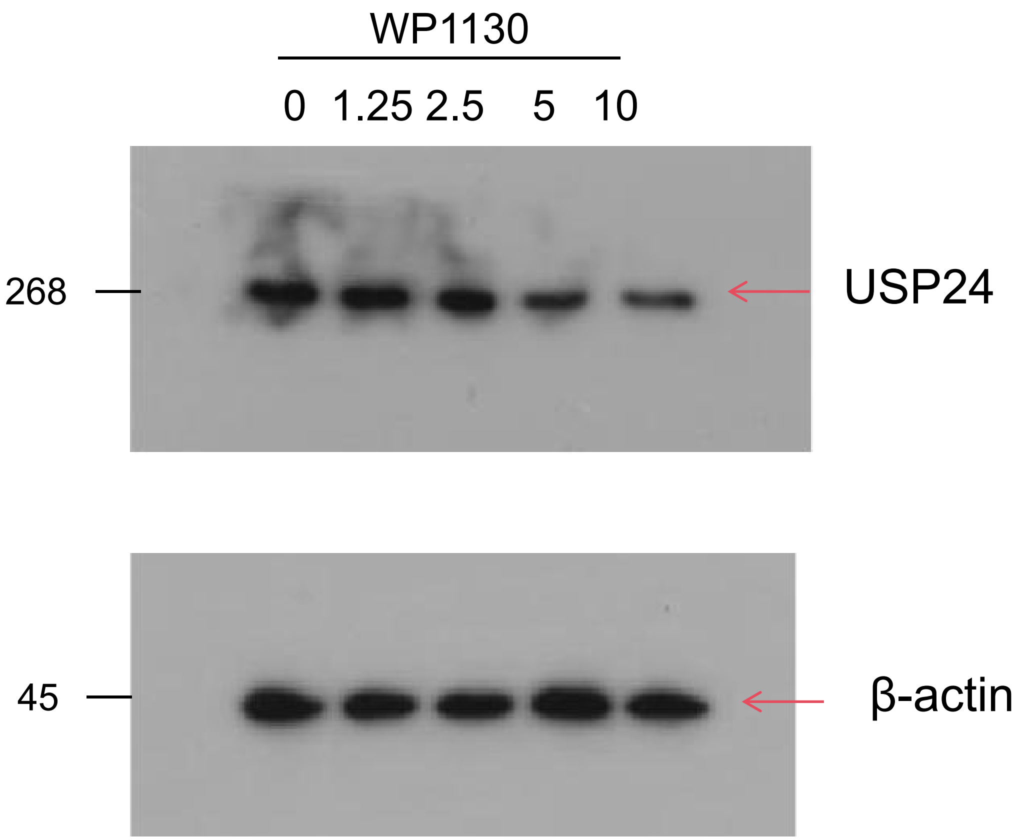

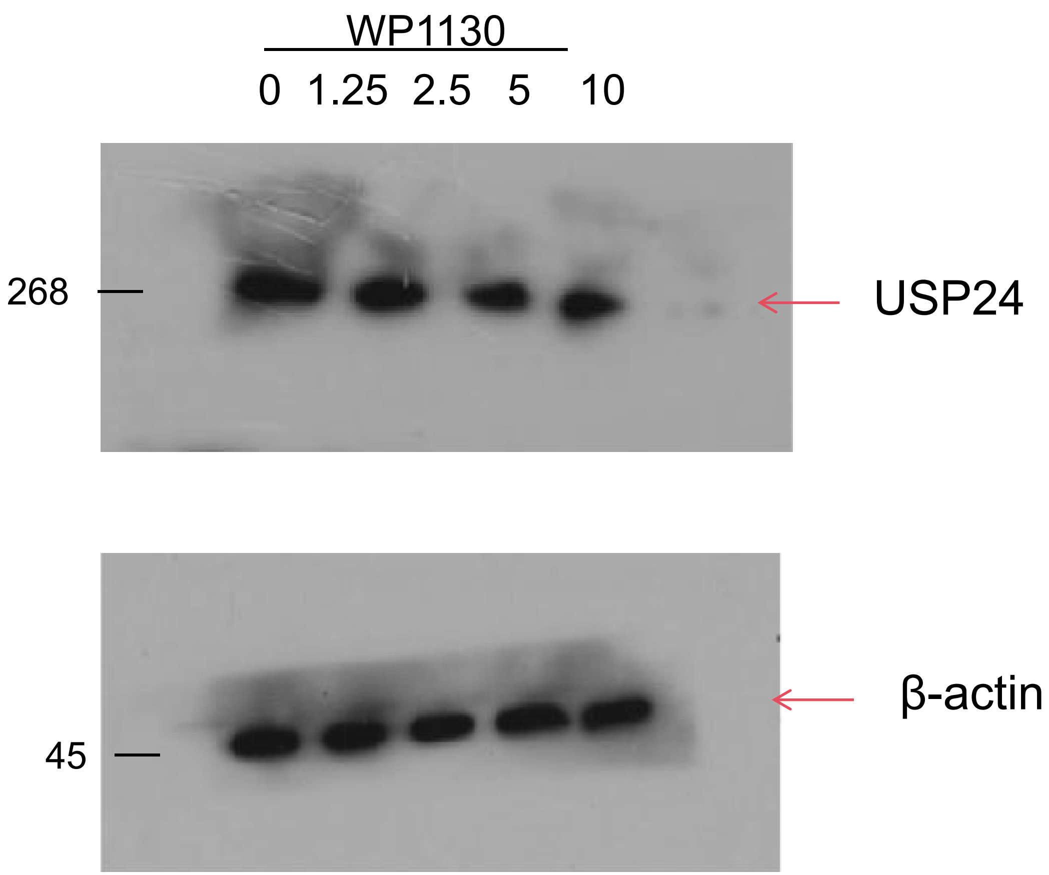

Original image:Fig.4E

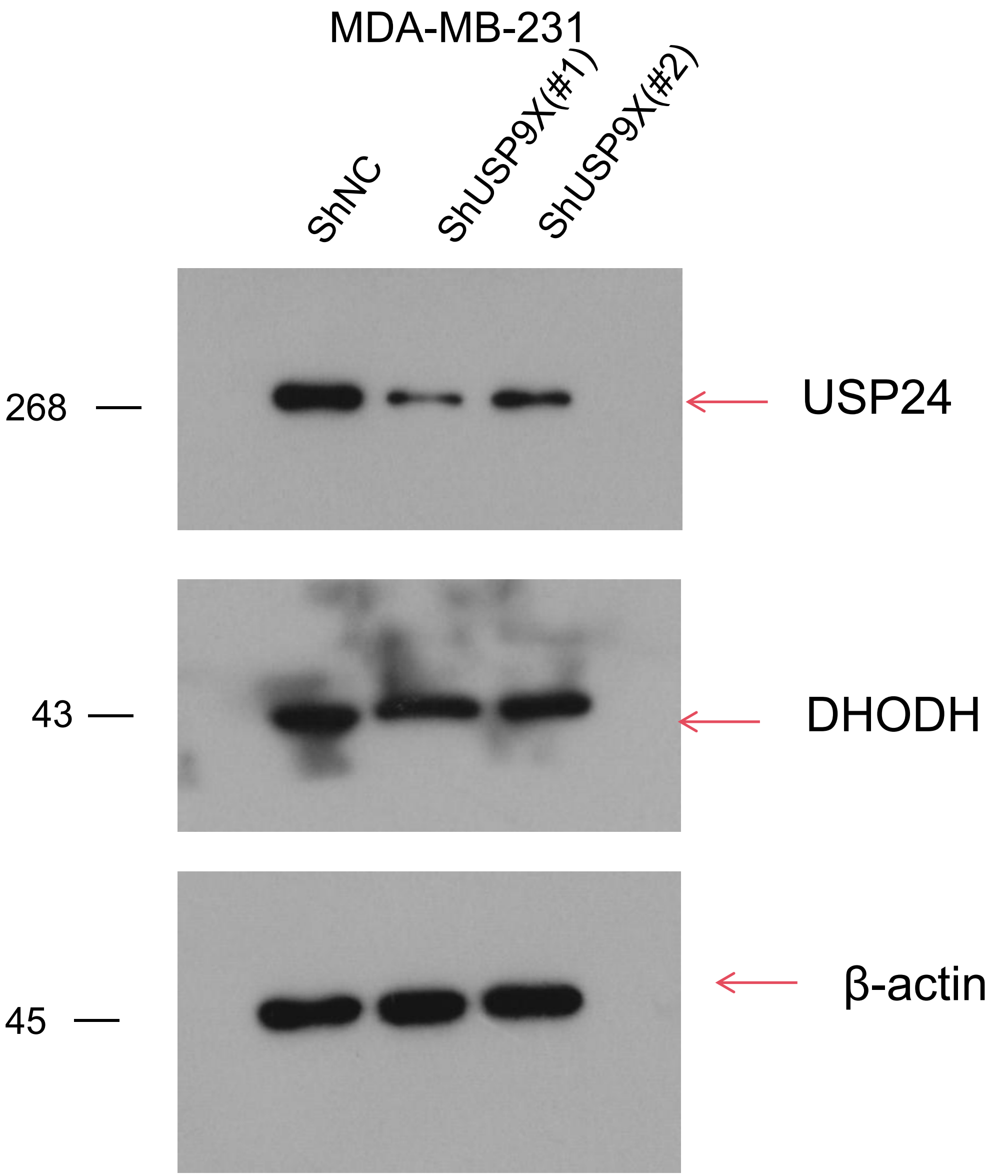

Original image: Fig.4G

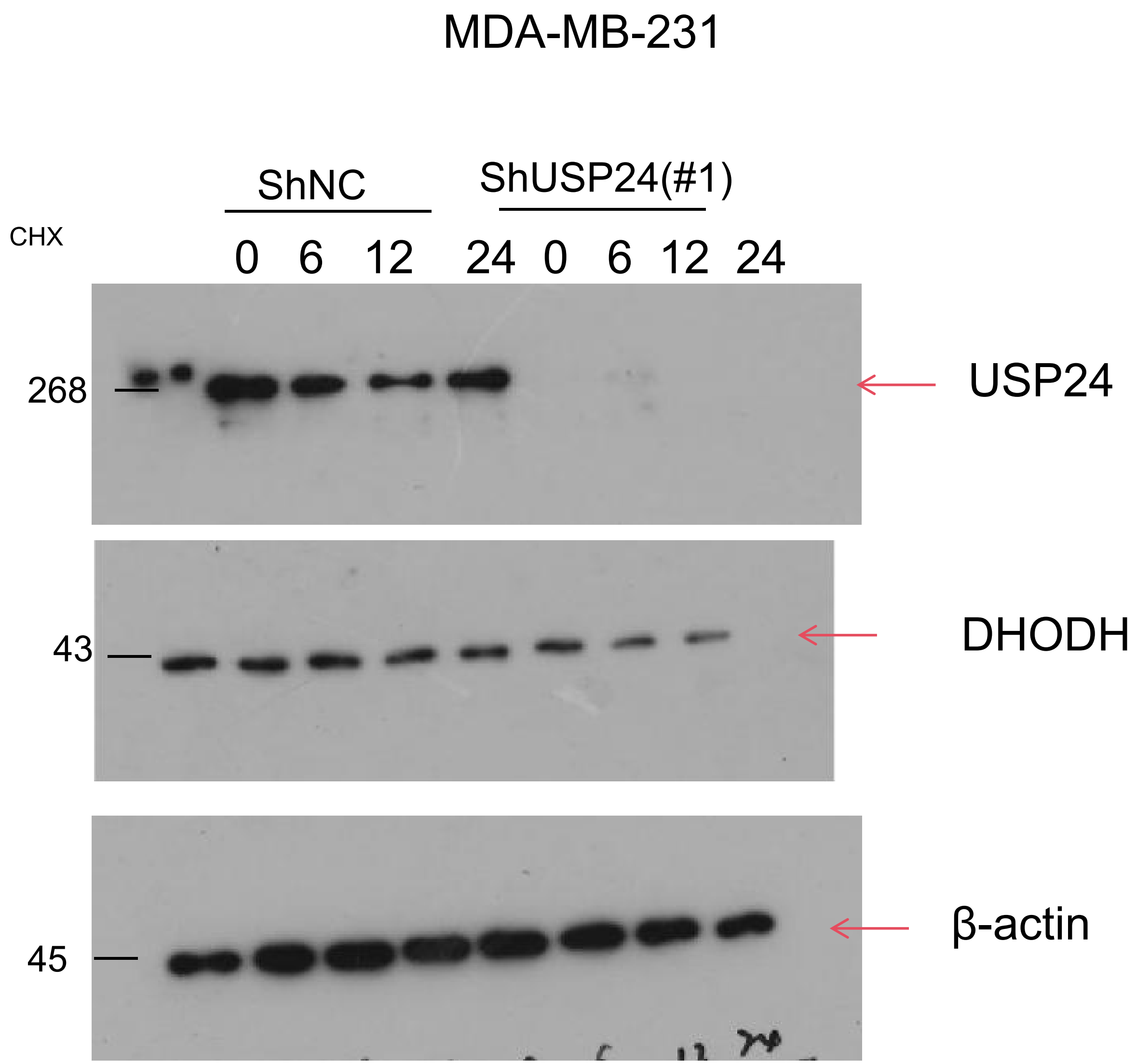

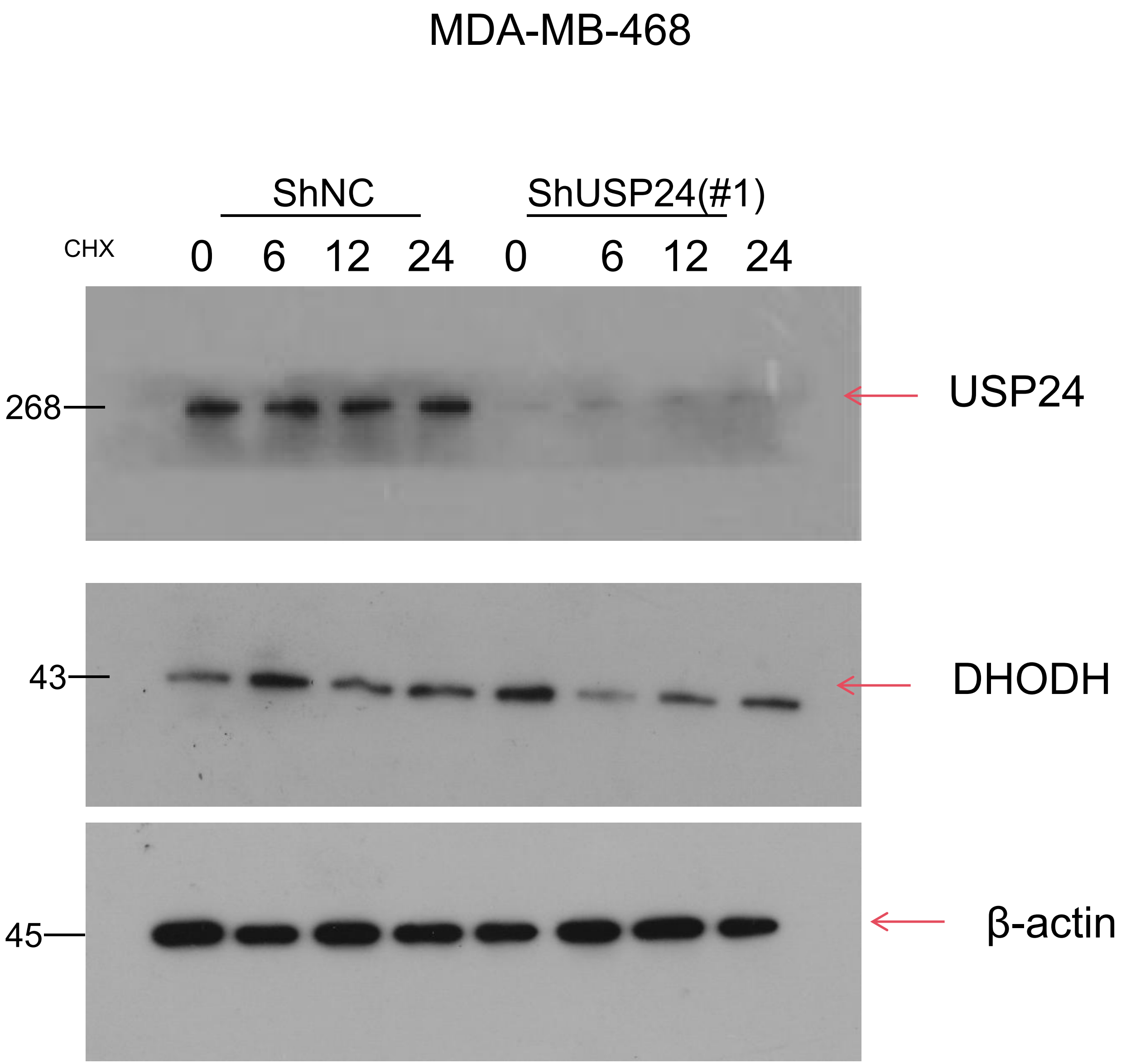

Original image: Fig.4I

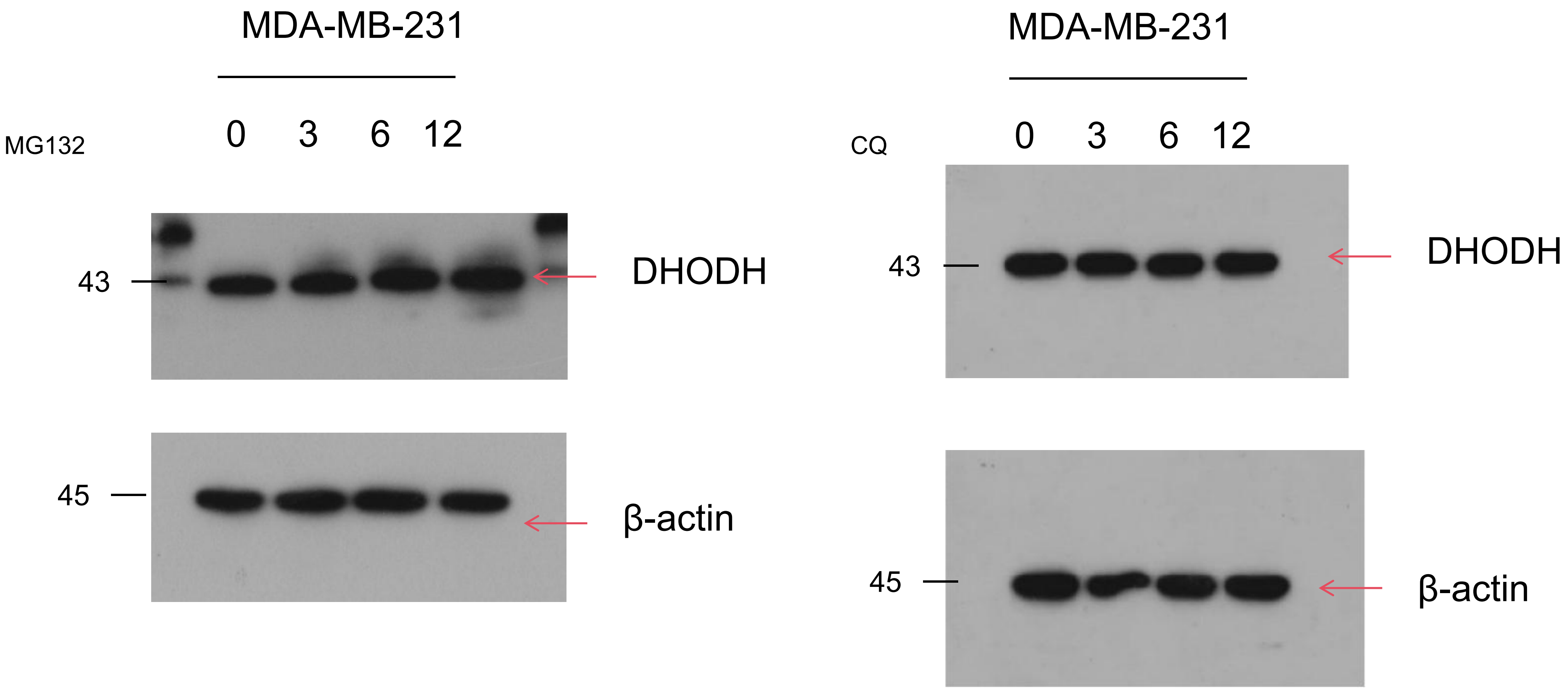

Original image: Fig.4K

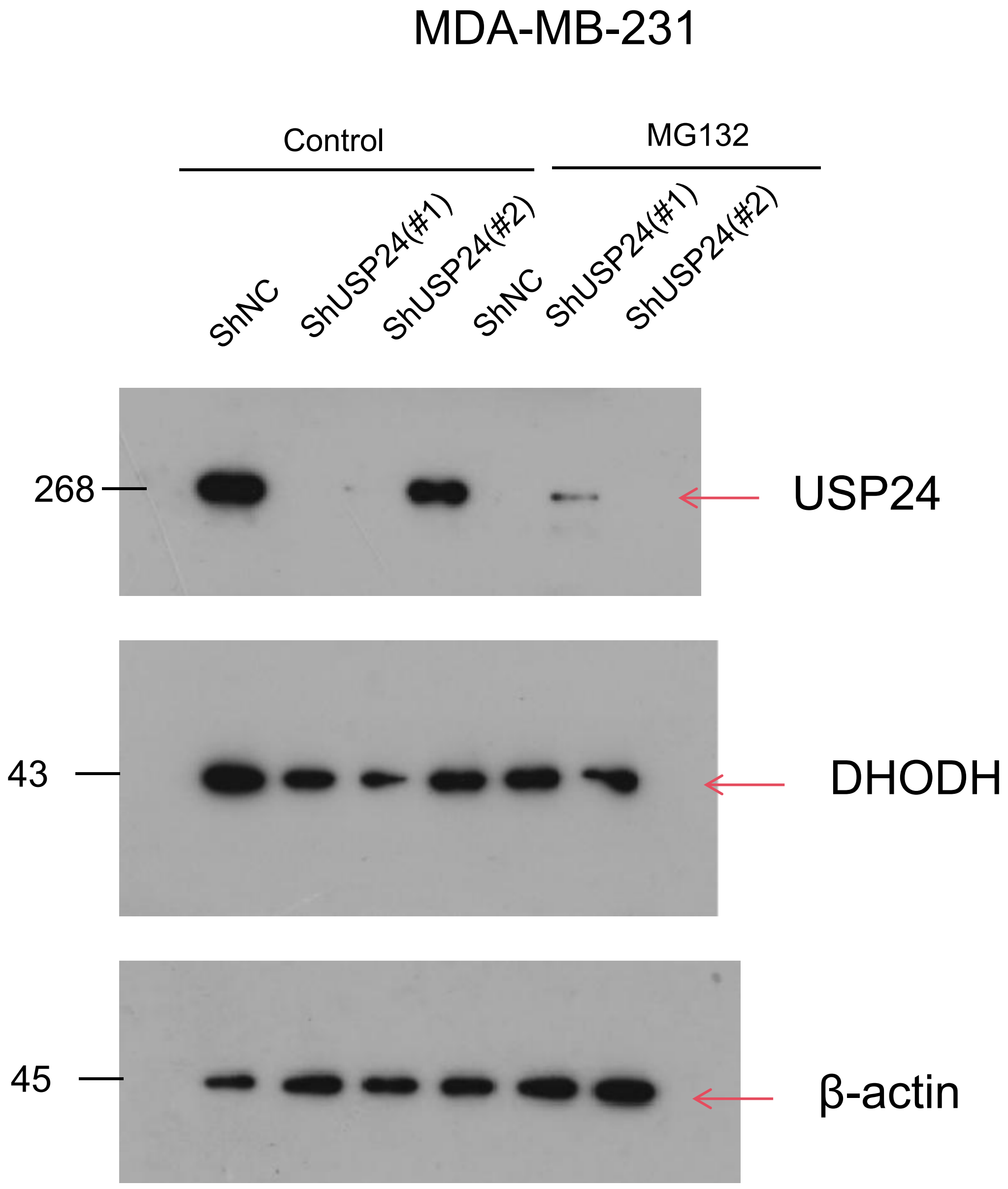

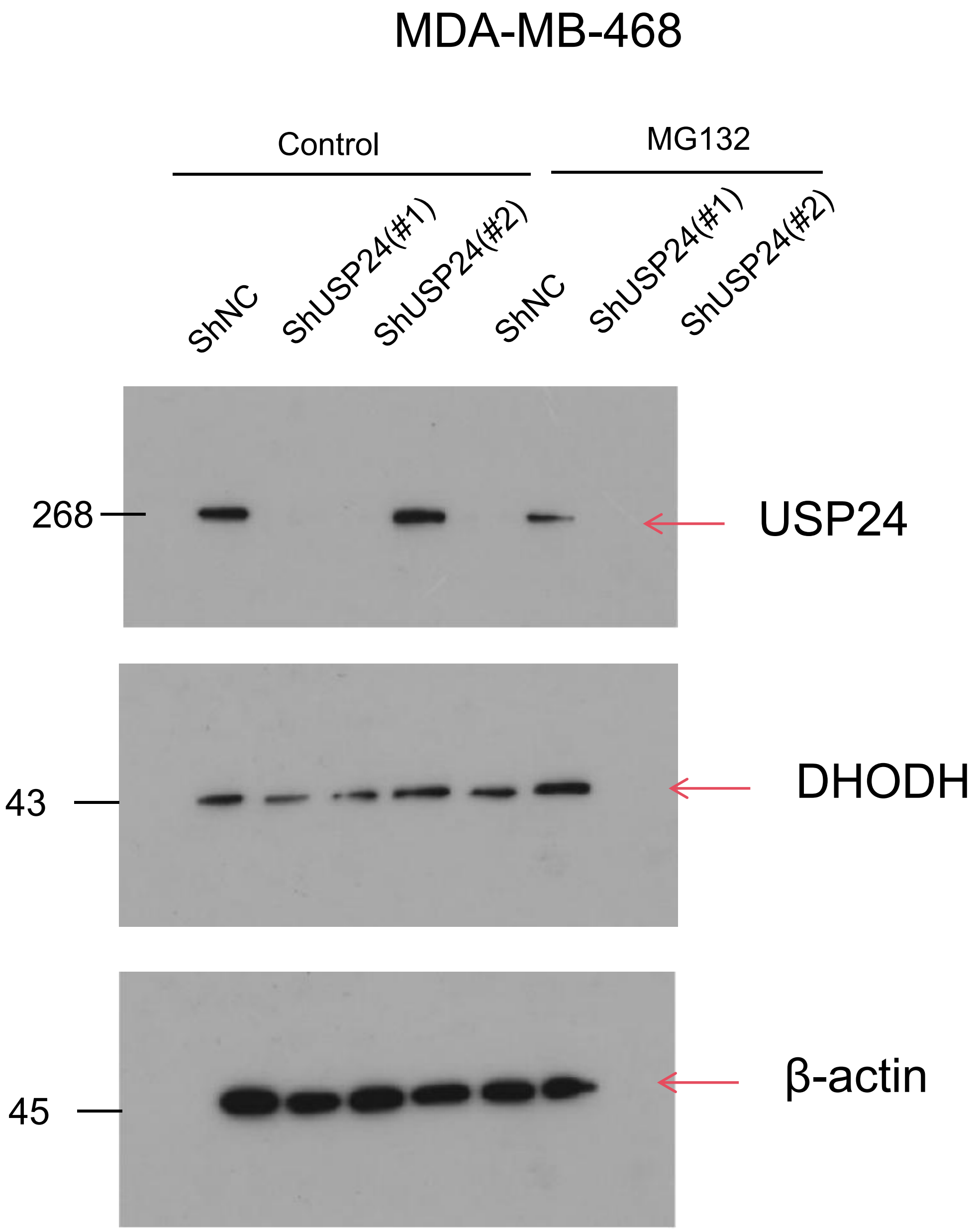

Original image: Fig.5A

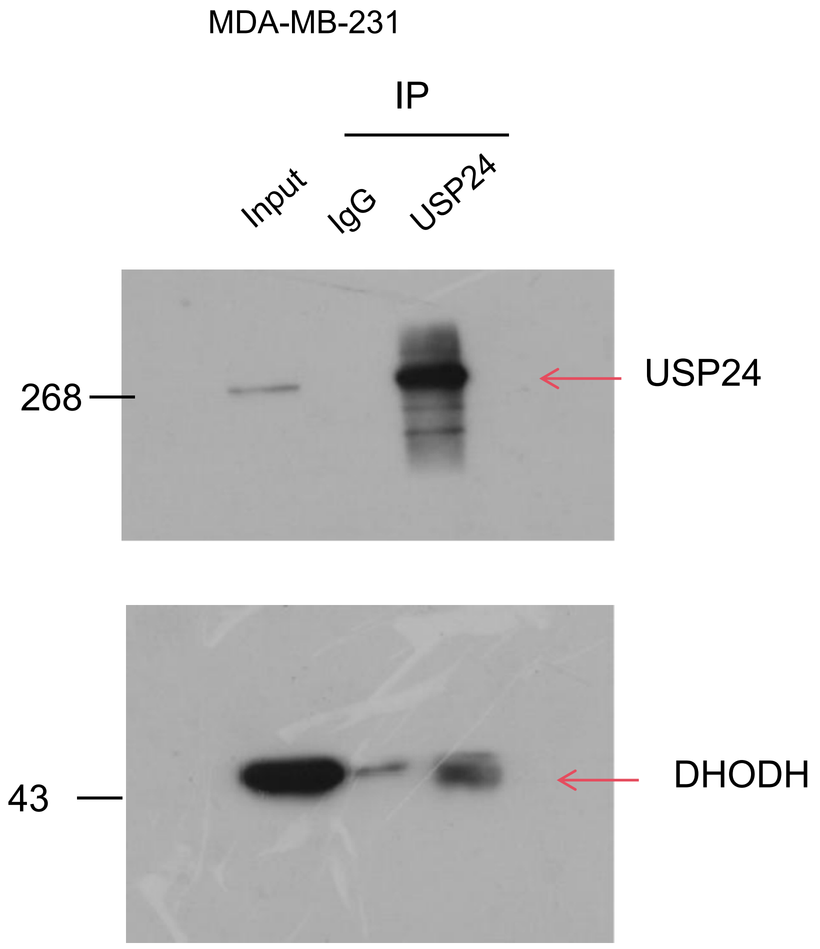

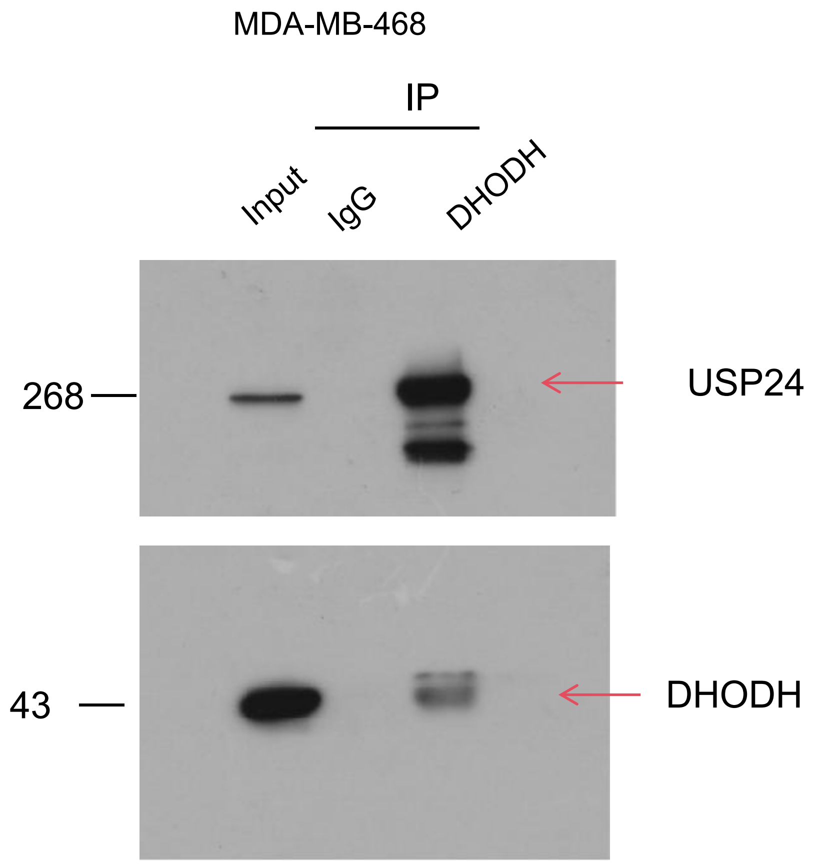

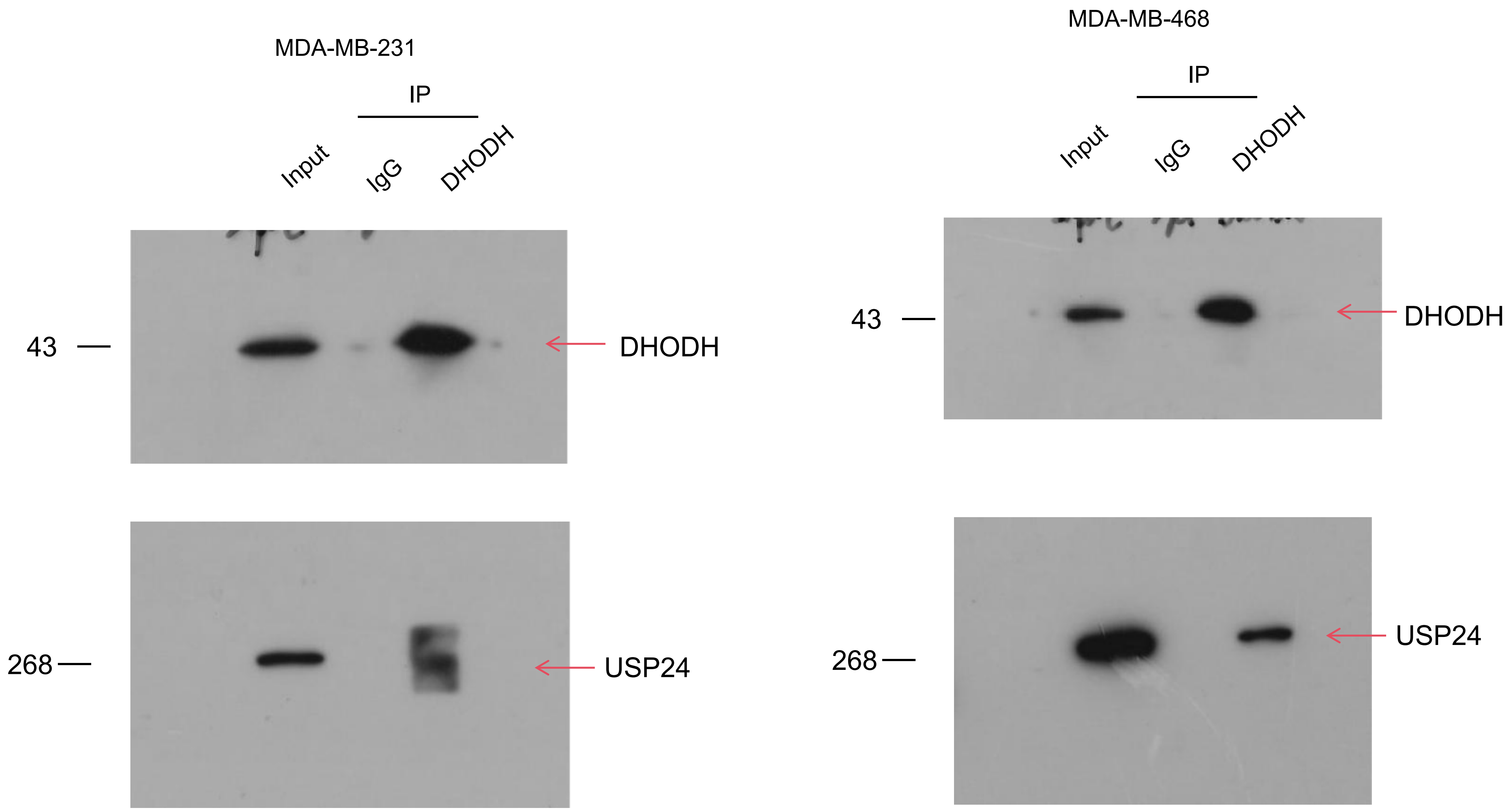
MDA-MB-231
IP
Input
IgG
DHODH
43
DHODH
268
USP24
MDA-MB-468
IP
Input
IgG
DHODH
43
DHODH
268
USP24

Original image: Fig.5E

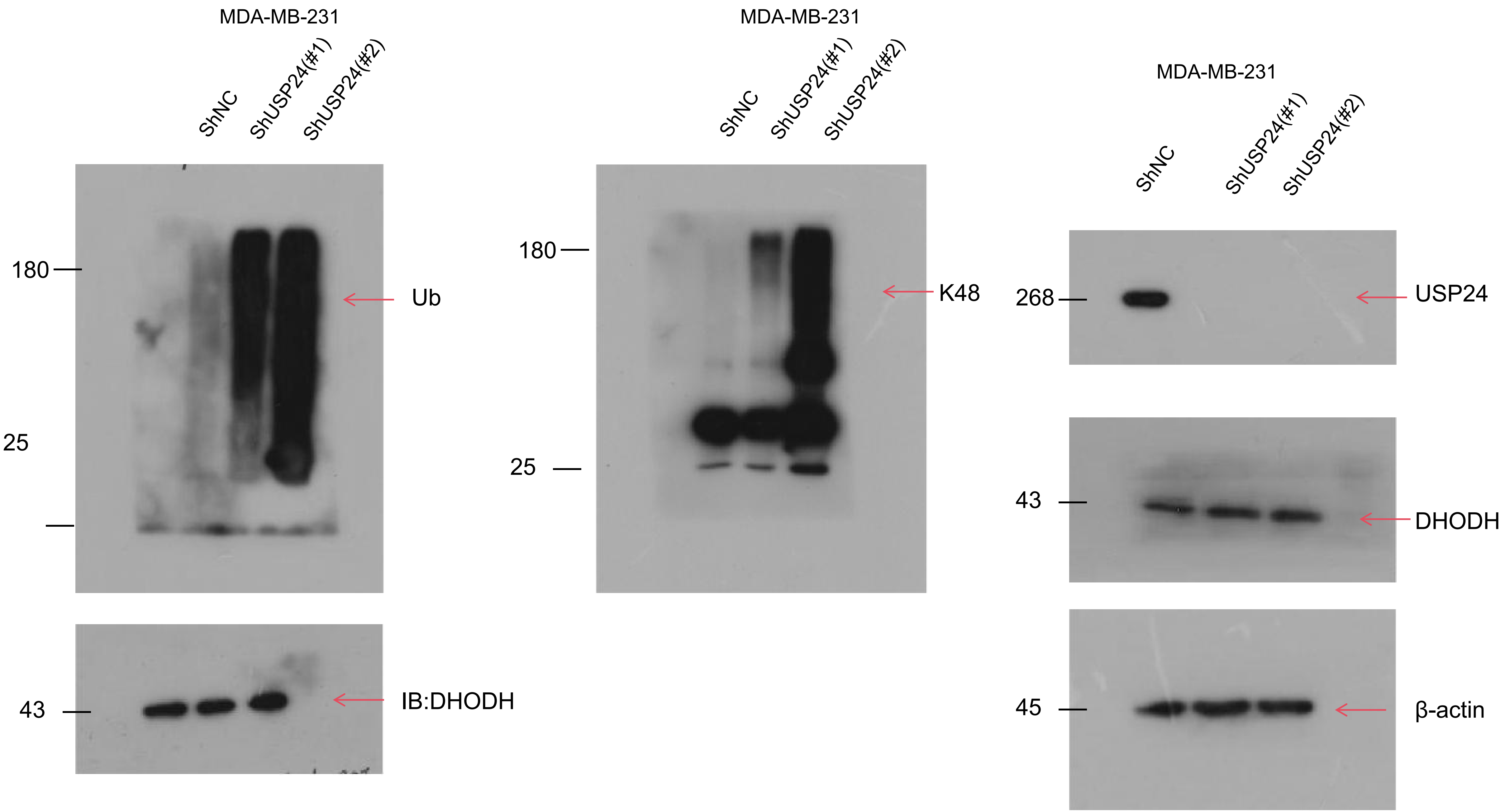

Original image: Fig.5G

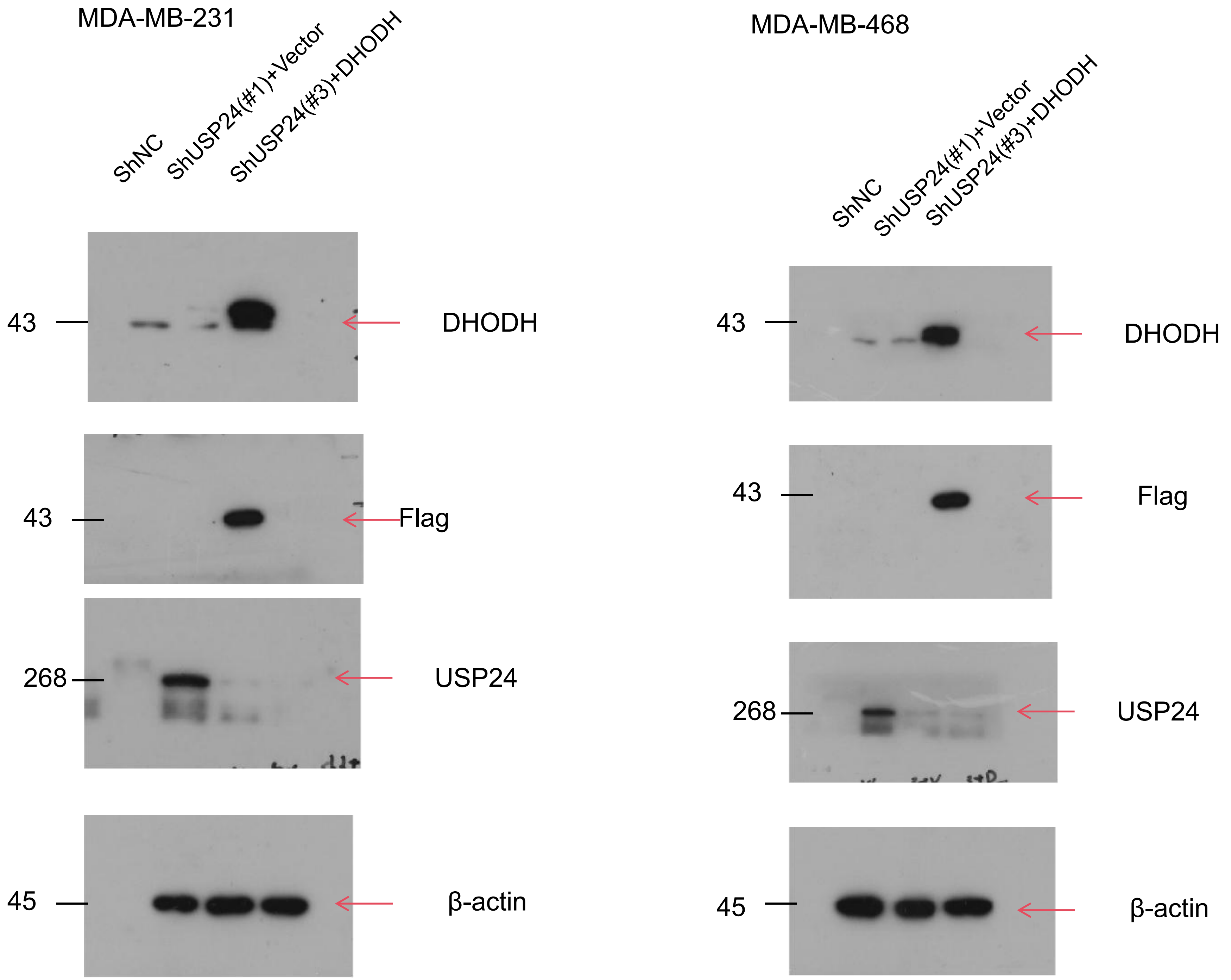

Supplement: Supplementary file 2 — Uncropped western blot images [file 41419_2025_7895_MOESM2_ESM.pdf]
